# Natural Functional SNPs in *miR-155* Alter Its Expression Level, Blood Cell Counts, and Immune Responses

**DOI:** 10.3389/fimmu.2016.00295

**Published:** 2016-08-02

**Authors:** Congcong Li, Huabin He, An Liu, Huazhen Liu, Haibo Huang, Changzhi Zhao, Lu Jing, Juan Ni, Lilin Yin, Suqin Hu, Hui Wu, Xinyun Li, Shuhong Zhao

**Affiliations:** ^1^Key Laboratory of Agricultural Animal Genetics, Breeding, and Reproduction of the Ministry of Education, Huazhong Agricultural University, Wuhan, China; ^2^Key Laboratory of Swine Genetics and Breeding of the Ministry of Agriculture, Huazhong Agricultural University, Wuhan, China; ^3^College of Animal Science and Technology, Henan University of Animal Husbandry and Economy, Zhengzhou, China; ^4^The Cooperative Innovation Center for Sustainable Pig Production, Wuhan, China

**Keywords:** *miR-155*, natural functional SNP, expression level, immune response, blood parameters

## Abstract

*miR-155* has been confirmed to be a key factor in immune responses in humans and other mammals. Therefore, investigation of variations in *miR-155* could be useful for understanding the differences in immunity between individuals. In this study, four SNPs in *miR-155* were identified in mice (*Mus musculus*) and humans (*Homo sapiens*). In mice, the four SNPs were closely linked and formed two *miR-155* haplotypes (A and B). Ten distinct types of blood parameters were associated with *miR-155* expression under normal conditions. Additionally, 4 and 14 blood parameters were significantly different between these two genotypes under normal and lipopolysaccharide (LPS) stimulation conditions, respectively. Moreover, the expression levels of *miR-155*, the inflammatory response to LPS stimulation, and the lethal ratio following *Salmonella typhimurium* infection were significantly increased in mice harboring the AA genotype. Further, two SNPs, one in the loop region and the other near the 3′ terminal of pre-*miR-155*, were confirmed to be responsible for the differential expression of *miR-155* in mice. Interestingly, two additional SNPs, one in the loop region and the other in the middle of miR-155*, modulated the function of *miR-155* in humans. Predictions of secondary RNA structure using RNAfold showed that these SNPs affected the structure of *miR-155* in both mice and humans. Our results provide novel evidence of the natural functional SNPs of *miR-155* in both mice and humans, which may affect the expression levels of mature *miR-155* by modulating its secondary structure. The SNPs of human *miR-155* may be considered as causal mutations for some immune-related diseases in the clinic. The two genotypes of mice could be used as natural models for studying the mechanisms of immune diseases caused by abnormal expression of *miR-155* in humans.

## Introduction

MicroRNAs (miRNAs) are small non-coding RNAs that inhibit the expression of specific target genes at the posttranscriptional level. Mature miRNAs are produced in a two-step sequential process that involves the generation of pre-miRNA from pri-miRNA *via* processing by the Drosha/DGCR8 complex in the nucleus, followed by the generation of mature miRNAs from pre-miRNAs *via* processing by the Dicer/TRBP complex in the cytoplasm. Sequence variations in pri-miRNAs, pre-miRNAs, and mature miRNAs may have profound effects on miRNA biogenesis and function (1).

*miR-155* is derived from the non-coding transcript of the proto-oncogene *B-cell integration cluster* (*bic*) and is highly expressed in activated B and T cells as well as active macrophages and dendritic cells (DCs) (2). Many studies have confirmed that *miR-155* plays important roles in the development and activation of immune-related cells. Overexpression of *miR-155* in normal human CD34^+^ peripheral blood stem cells (PBSCs) significantly inhibited the generation of myeloid and erythroid colonies (3). In *miR-155*-deficient mice, the number of natural killer cell is significantly reduced compared with wild-type mice (4). Additionally, the amount of IgG1 produced by B cells following stimulation with lipopolysaccharide (LPS) and *interleukin 4* (*IL-4*) was significantly reduced by *miR-155* deficiency (4). Moreover, Th2 polarization and Th2 cytokine levels were significantly increased in CD4^+^ T cells derived from *miR-155*-deficient mice (4, 5). Fewer Th17 and Th1 cells were present in the spleen and lymph nodes under experimental autoimmune encephalomyelitis in *miR-155*-deficient mice (6). *miR-155*-deficient DCs failed to efficiently present antigens (4). The abundance of T regulatory cells was significantly reduced in the thymus, spleen, and lymph nodes of *miR-155*-deficient mice (7). In addition, *miR-155* deficiency impairs CD8^+^ T cell proliferation (2), and *miR-155* is essential for promoting the clonal expansion, survival, and memory generation behavior of CD8^+^ T cells during antiviral and antibacterial responses (8). Many target genes of *miR-155* have been identified, and most of these genes are essential to hematopoietic development. Previous studies have shown that *miR-155* can directly repress the *C/EBP*β, *PU.1, SHIP1, Tab2, Ikbke, map3k14*, and *Bach1* genes (9–13). These studies indicate that *miR-155* plays crucial roles in immune cell development and immune responses.

In this study, four SNPs were identified in humans and mice. The roles of these SNPs in *miR-155* expression and the immune response were further investigated. Two functional SNPs were identified in both humans and mice; these SNPs were responsible for altering the expression levels of mature *miR-155* and modulating *miR-155*-mediated immune responses. Our findings provide new evidence of the functional SNPs of *miR-155* and their effects on *miR-155*-regulated immune responses.

## Materials and Methods

### Animals

C57BL/6 and Kunming mice were purchased from the Center for Disease Control of Hubei Province, China. The animals were housed five per cage at room temperature, with a 12-h light/dark cycle and free access to water and food. This study used 5- to 6-week-old mice. The experiments were performed in accordance with the Guide for the Care and Use of Laboratory Animals (14), and the protocols received approval from the Hubei Province Committee on Laboratory Animal Care (HZAUMU2013-0005).

### LPS Administration and *Salmonella typhimurium* Infection

Purified LPS extract from *S. typhimurium* (Sigma, L6511) was dissolved in sterile phosphate-buffered saline (PBS) at 1 mg/ml and frozen in aliquots at −20°C. Animals were administered LPS *via* intraperitoneal injection at 10 mg/kg according to previous studies (15–17). Animals were infected with 5 × 10^6^ colony-forming units (CFU) of *S. typhimurium via* intraperitoneal injection.

### Identification of *miR-155* Polymorphisms

For identification of polymorphism in human *miR-155*, the data of 1,000 genome sequence were examined (18, 19). Four candidate SNPs in the pre-*miR-155* were selected for functional evaluation. In mice, the 256-bp DNA fragment containing the mature *miR-155* sequence was amplified using DNA samples from C57BL/6 and Kunming mice. Polymerase chain reaction (PCR) was performed in 10-μl reactions containing 10 × PCR buffer, 0.3 μM of each primer, 75 μM of dNTPs, 1 U of Taq DNA polymerase (Takara Biotechnology), and 50 ng of mouse genomic DNA. The PCR cycling conditions were 5 min at 94°C, followed by 36 cycles of 30 s at 94°C, 30 s at 60°C, and 20 s at 72°C and a final extension step of 5 min at 72°C. The polymorphisms were identified based on the sequencing results. Ultimately, four SNPs were identified in mice, and these SNPs were closely linked and formed two haplotypes.

### Analysis of the Associations of Hematological Parameters

A total of 552 mice from 53 crosses (AB × AB) were used for correlation analysis. Because one of the SNPs in the haplotype could be detected using the restriction enzyme *Stu*I, the genotype of the population was determined using the PCR-restriction fragment length polymorphism (RFLP) method. For phenotype detection, peripheral blood from each mouse at 5–6 weeks of age was collected into an anticoagulant vacuum blood tube. A total of 24 blood parameters were measured using a hematological analysis system (Sysmex XT-2000i, Sysmex, Japan); the tested parameters included white blood cell count (WBC), absolute neutrophil count (NEUT), absolute lymphocyte count (LYMPH), absolute monocyte count (MONO), absolute eosinophil count (EO), absolute basophil count (BASO), neutrophil percentage (NEUTp), lymphocyte percentage (LYMPHp), monocyte percentage (MONOp), eosinophil percentage (EOp), basophil percentage (BASOp), red blood cell count (RBC), hemoglobin concentration (HGB), hematocrit (HCT), mean cell volume (MCV), mean corpuscular hemoglobin (MCH), mean corpuscular hemoglobin concentration (MCHC), red cell distribution width-SD (RDW-SD), red cell distribution width-CV (RDW-CV), platelet count (PLT), plateletcrit (PCT), mean platelet volume (MPV), platelet distribution width (PDW), and platelet-large cell ratio (PLCR). Moreover, 52 and 43 mice harboring the AA and BB genotype, respectively, were selected for analysis of the variations in blood parameters in response to LPS treatment. The mice were intraperitoneally injected with 10 mg/kg LPS. After 8 h of LPS stimulation, hematological parameters were measured. Analysis of the associations between genotypes and blood parameters was performed using the following GLM model in SAS (SAS Institute, Inc., Cary, NC, USA): *Y* = genotype + sex + e; sex was considered as a fix effect in this model.

### Northern Blotting

For northern blotting analysis, total RNA was extracted using TRIzol reagent according to the manufacturer’s protocol (Invitrogen, USA). From 15 to 30 μg of total RNA was electrophoretically separated on a 15% polyacrylamide denaturing gel. The total RNA was then transferred to a Hybond-N+ membrane (Amersham Biosciences, UK) using a semidry Transblot electrophoresis apparatus (Bio-Rad, USA). The RNA was fixed to the membrane by heating the membrane in the oven at 80°C for 30 min and then cross-linked to the membrane *via* UV irradiation. Hybridization was performed using PerfectHyb™ Hybridization Solution according to the manufacturer’s protocol (Toyobo, Japan). The hybridization probe sequence was complementary to the mature form of *miR-155* (Table S1 in Supplementary Material) and was labeled with γ-^32^P. After washing, the membranes were imaged using a phosphor imager (Bio-Rad, USA). *U6* was used as a control and was detected in a manner similar to *miR-155*. The signals of the northern blotting bands were quantified using Quantity One software (Bio-Rad, USA).

### Tissue Sections

Spleen, lung, and liver tissues were collected from Kunming mice harboring the AA or BB genotype following LPS treatment or no treatment. After 24 h of fixation, the spleen, lung, and liver tissues were dehydrated and embedded in paraffin wax. Then, 4-μm tissue sections were sliced using a microtome (Leica Microsystems Nussloch GmbH, Nussloch, Germany), mounted on poly-lysine-coated slides (Boster, Wuhan, China), and stored at 4°C until staining. Afterward, the sections were deparaffinized in xylene, rehydrated in a graded ethanol series, and stained with hematoxylin and eosin. Finally, the sections were mounted using neutral gum. Staining was examined *via* light microscopy (Olympus BX51, Tokyo, Japan).

### Enzyme-Linked Immunosorbent Assay

To compare the cytokine levels between the two genotypes, we first collected serum from peripheral blood. In brief, whole blood from the eye socket of each mouse was collected into clean tubes. The tubes were then placed at an angle of 45° to 60° and incubated for 1 h at 37°C. Then, the tubes were centrifuged at 3,000 rpm for 5 min at room temperature. The supernatant was transferred to a new tube and centrifuged further at 12,000 rpm for 2 min at room temperature. Then, the purified supernatant was stored at −80°C. For cytokine (*IL1*β, *TNF*α, *IL6*, and *IL8*) detection, mouse ELISA kits were used, and all of the experimental steps were performed strictly according to the manufacturer’s protocol (NeoBioscience, Guangzhou, PR China). The fluorescence at 450 nm was measured using a microplate reader (iMark, Bio-Rad, USA). The concentration of each cytokine present in the samples was calculated in reference to a standard curve that was constructed using recombinant cytokines provided with each kit.

### RNA-Seq Analysis

Total RNA was obtained from spleen tissues after 0, 4, and 8 h of LPS treatment using TRIzol reagent (Invitrogen, USA). For each genotype, equal amounts of RNA from three individuals at each time point were pooled together. Ultimately, six RNA libraries were generated, and RNA-seq was performed by a commercial service (Genergy Biotechnology, Shanghai, PR China). The mRNA sequencing data were analyzed using Bowtie, TopHat, and Cufflinks (20). The reference genome version was GRCm38/mm10.

### Quantitative PCR

After determining the RNA concentrations, reverse transcription was performed using a RevertAid First Strand cDNA Synthesis Kit (MBI Fermentas). For quantitative PCR (Q-PCR) analysis of *miR-155*, stem–loop RT primers were designed according to a previous report (21). Q-PCR was performed using a standard SYBR Green PCR kit (Toyobo, Japan) in a LightCycler 480 thermal cycler (Roche, Switzerland). The PCR protocol was 2 min at 95°C followed by 40 cycles of amplification (30 s at 95°C, 30 s at the annealing temperature, and 30 s at 72°C). Melting curves were obtained by increasing the temperature from 58 to 95°C at 0.5°C/s and then holding for 10 s. The *U6* or *tubulin* gene was used as an internal control for the genes expression of *miR-155, SHIP1*, and *PU.1*. PCR was performed at least in triplicate, and relative gene expression levels were calculated using an optimized comparative Ct (ΔΔCt) method. The primers used for Q-PCR detection are listed in Table S1 in Supplementary Material. The significance of the differences in gene expression was analyzed using a *t*-test.

### Western Blotting

To confirm the differential protein expression of the two important hematopoietic-related *miR-155* target genes between the two genotypes of mice, spleen tissue extracts were fractionated according to molecular weight *via* sodium dodecyl sulfate-polyacrylamide gel electrophoresis (SDS-PAGE) and then transferred to a polyvinylidene difluoride (PVDF) membrane using a semidry transfer apparatus (Bio-Rad, USA). Western blotting was performed using the following antibodies: anti-*PU.1* (9G7), anti-*SHIP1* (D1163), anti-*GAPDH* (14C10), and horseradish peroxidase (HRP)-conjugated anti-rabbit IgG (Cell Signaling Technology, USA). All of the antibodies were diluted 1,000-fold. The signal of the western blotting bands was quantified using ImageJ software (National Institutes of Health, USA).

### DNA Construction of Different *miR-155* SNPs

The 256-bp mouse *miR-155* expression cassette containing the entire pre-*miR-155* sequence and partial flanking regions was inserted into the 3′ untranslated region (UTR) of the green fluorescent protein (GFP) gene in the pEGFP-C1 vector (BD Biosciences Clontech, USA). The two haplotypes (*A* and *B*) and seven mutant constructs (M1–M7) were produced and were labeled as pEGFP-C1-A/B/M1–M7. The 255-bp human *miR-155* fragments were inserted into the 3′UTR of the GFP gene as performed on mouse *miR-155*. The normal and four mutant constructs of human *miR-155* gene were generated and were labeled as pEGFP-C1-h or pEGFP-C1-h1–4, respectively. For the luciferase assay, the 3′UTR fragments of the human and mouse *Tab2, Bach1, Ikbke*, and *Map3k14* genes were inserted into the psi-check2 vector. The inserted fragments contained the validated *miR-155* binding sites located at the 3′UTR of the respective target genes.

### Cell Culture and Transfection

BHK-21 cells were cultured in complete Dulbecco’s modified eagle medium (DMEM) containing 10% fetal bovine serum (FBS) in a humidified incubator in 5% CO_2_ at 37°C. The different constructs were transfected at equal plasmid DNA concentrations into BHK-21 cells at 80–90% confluence. For analysis of *miR-155* expression *in vitro*, the pEGFP-C1-A, pEGFP-C1-B, pEGFP-C1-M1–M7, and pEGFP-C1 empty constructs were transfected into BHK-21 cells at equal plasmid DNA concentrations. For the luciferase activity assay, equal amounts of the pEGFP-C1-A, pEGFP-C1-B, pEGFP-C1-h, and pEGFP-C1-h1–4 constructs were co-transfected with one of the psi-check2-*Tab2/Bach1/Ikbke/Map3k14* vectors into BHK-21 cells.

### Statistical Analysis

All data are presented as the means ± SEM. Significant differences between groups were determined using a *t*-test or analysis of variance (ANOVA). A *P*-value <0.05 was considered to indicate a statistically significant difference, and a *P*-value <0.01 was considered to indicate an extremely significant difference.

## Results

### Identification of *miR-155* SNPs in Mice and Humans

**Figure 1:**
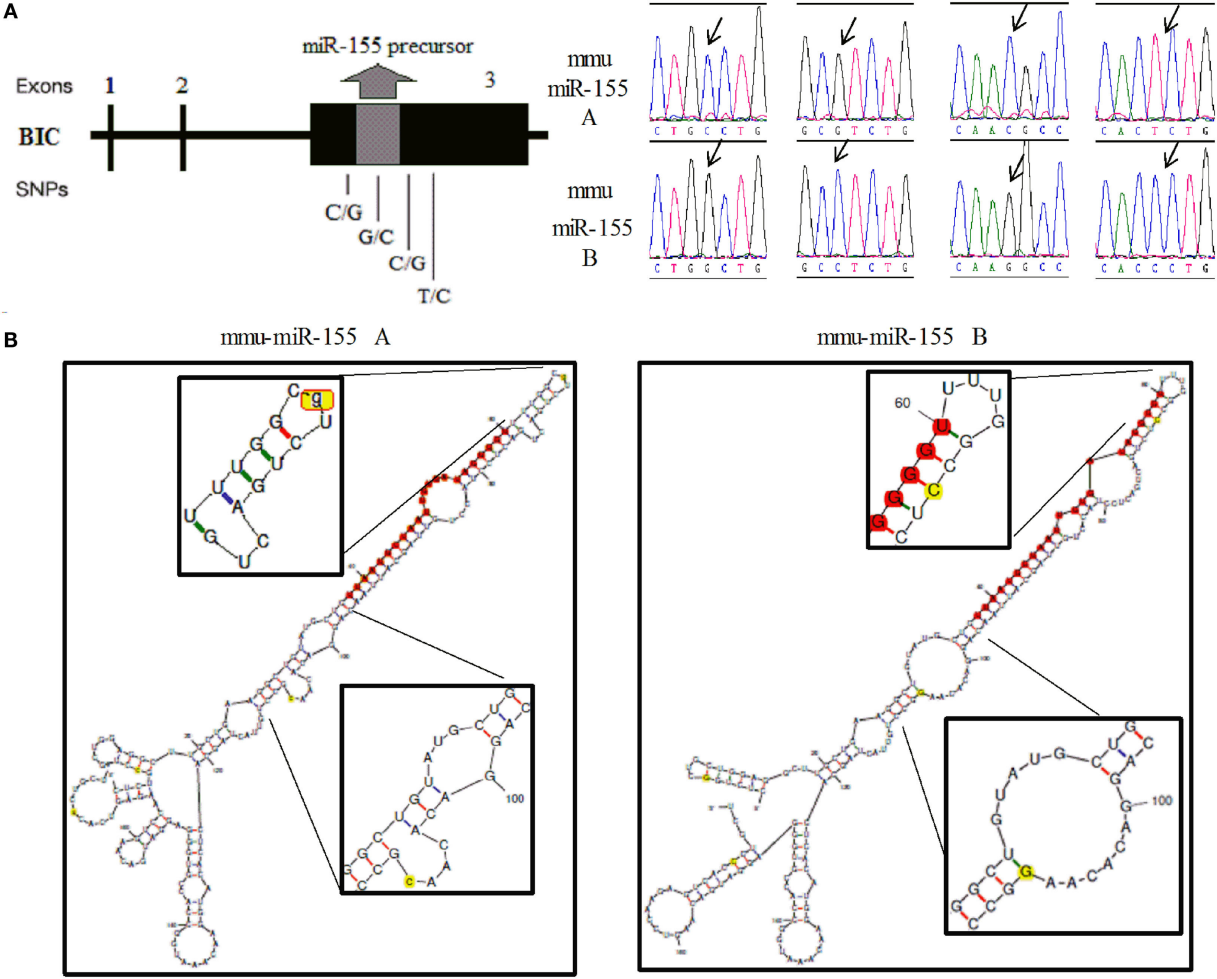
**Identification of the two haplotypes of the mouse *miR-155* gene and the corresponding secondary RNA structures**. **(A)** Schematic diagram of the SNP sites and sequences of the A and B haplotypes of the mouse *miR-155* gene. **(B)** Prediction of the secondary RNA structures of *miR-155* for the two haplotypes using mfold tools. The left image represents the A haplotype, and the right image represents the *B* haplotype. The SNP sites are highlighted in bright yellow. The mature *miR-155* sequences are highlighted in bright red. The secondary structure of the stem–loop and the Drosha digestion site are magnified.

**Figure 2:**
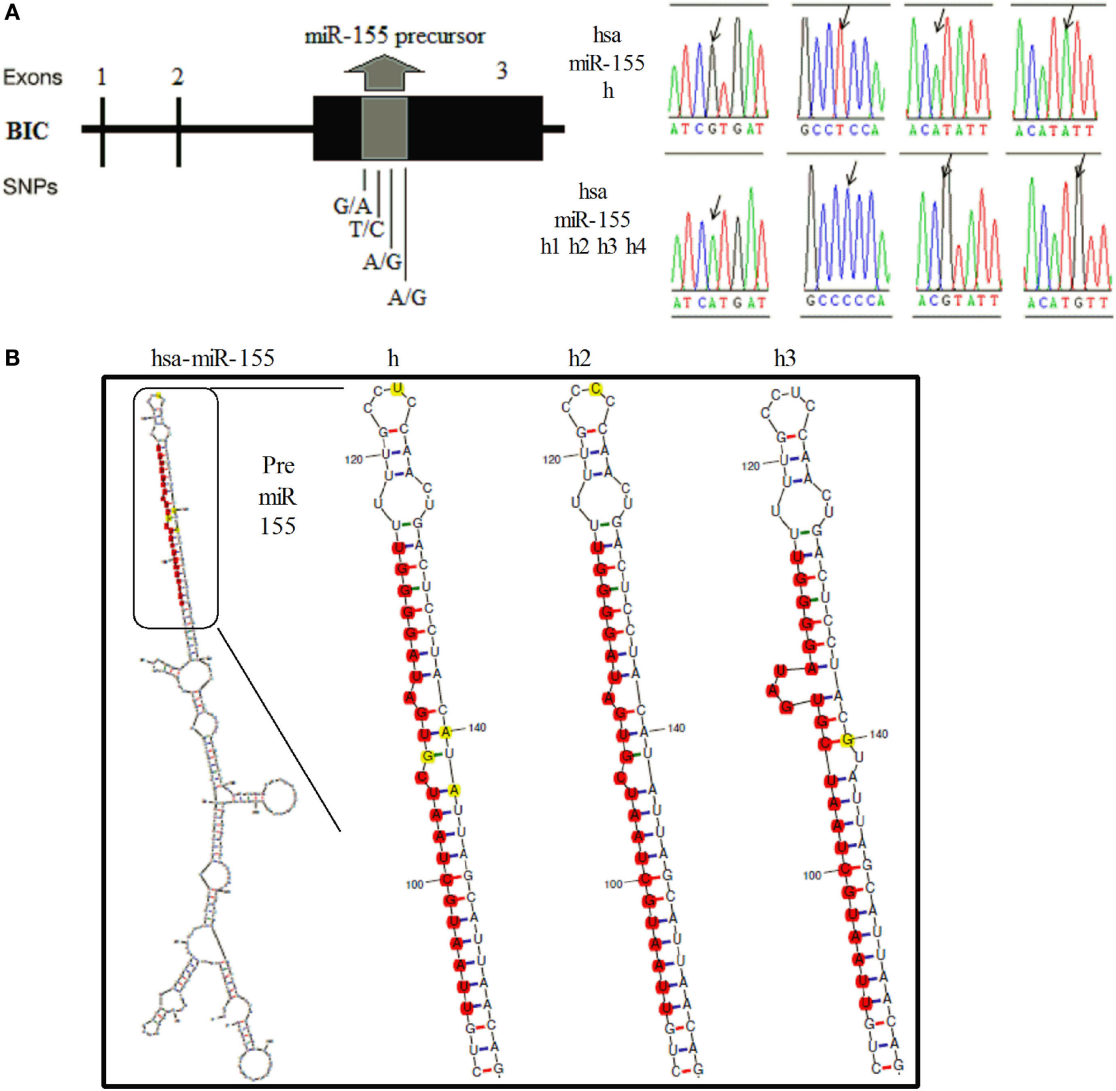
**Identification of SNPs of the human *miR-155* gene and the corresponding secondary RNA structures**. **(A)** Schematic diagram of the SNP sites and sequences of the human *miR-155* gene. **(B)** Prediction of the secondary RNA structures of human *miR-155* for the different SNPs using mfold tools. The SNP sites are highlighted in bright yellow. The mature *miR-155* sequences are highlighted in bright red.

In mice, a 256-bp genomic fragment of *miR-155* was amplified using DNA samples from non-biologically related Kunming (*n* = 25) and C57BL/6 mice (*n* = 20). The sequencing results for the different strains of mice revealed four SNPs in the 256-bp fragment, which formed two haplotypes (Figure 1A, Supplementary Figure 1). Furthermore, Kunming mice harbored the AA/AB and BB genotypes, but C57BL/6 mice harbored only the BB genotype. The secondary structures of *miR-155* containing different SNPs were predicted using mfold (http://unafold.rna.albany.edu/?q=mfold). According to these results, two SNPs in *miR-155*, one in the loop region of *miR-155* and the other near the 3′ end of pre-*miR-155*, influenced the secondary structure of pre-*miR-155* in mice (Figure 1B). In humans, according to results of 1,000 human genomic sequence, four SNPs located in the pre-*miR-155* regions were identified (Figure 2A, Supplementary Figure 2). The first SNP was located in the mature *miR-155* region, the second in the stem–loop regions of *miR-155*, and the third and the fourth in the miR-155* region. Of these four SNPs, only the third SNP changed the secondary structure of pre-*miR-155* (Figure 2B).

### Blood Parameters Significantly Differed between the AA and BB Genotypes of Kunming Mice

**Figure 3:**
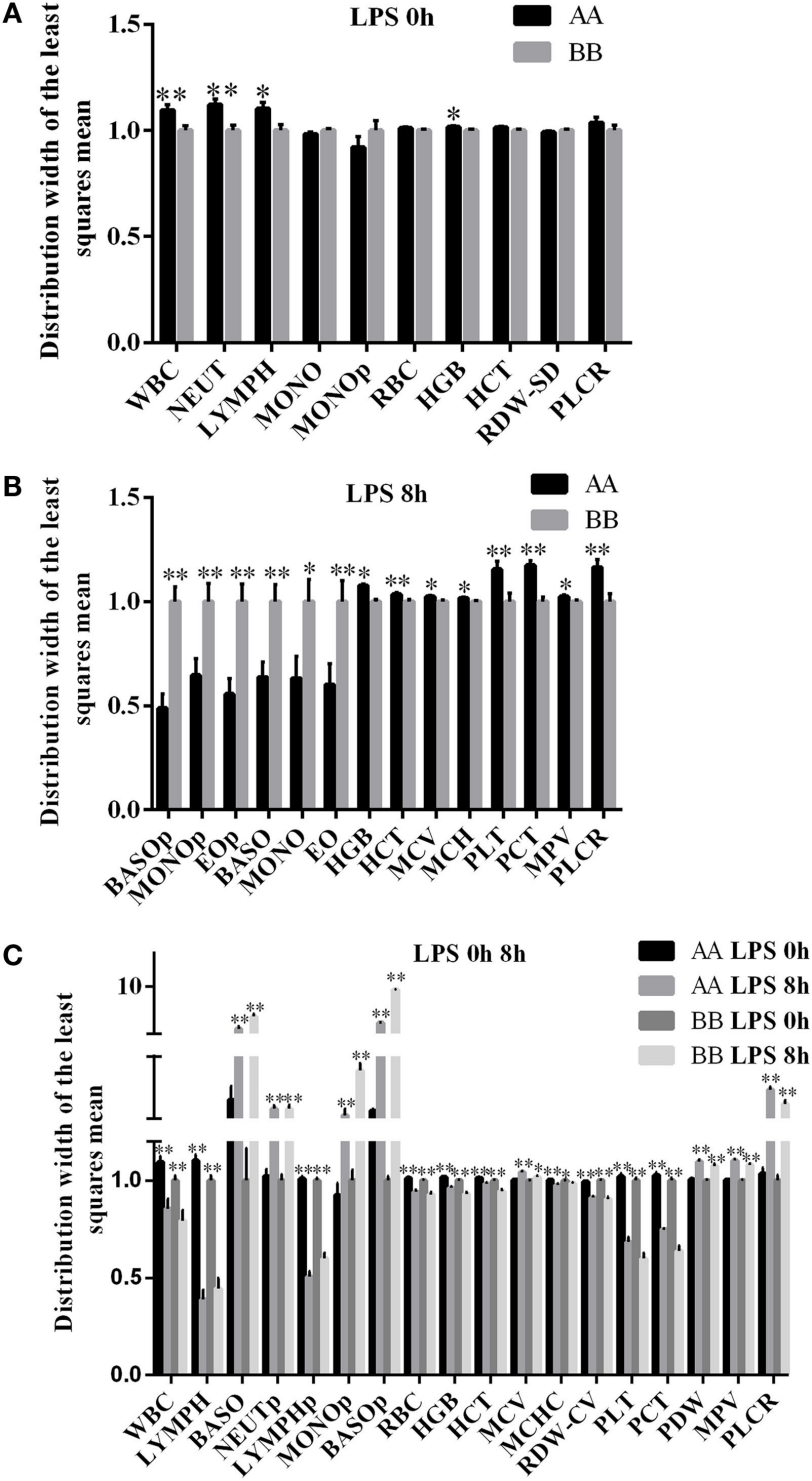
**Blood parameters in Kunming mice were significantly different between the two genotypes with or without LPS treatment**. **(A)** Trait association analysis indicated that 10 blood parameters were associated with the haplotypes of the mouse *miR-155* gene (*n* = 552; AA = 148; AB = 222; BB = 182). Among these blood parameters, four (WBC, NEUT, LYMPH, and HGB) were significantly increased in AA genotype mice compared with BB genotype mice under normal conditions. **(B)** A total of 14 blood parameters were significantly different between the AA and BB genotype mice after 8 h of LPS exposure (*n* = 95; AA = 52, and BB = 43). Among these blood parameters, six (BASOp, MONOp, BASO, MONO EO, and EOp) were significantly increased in BB genotype mice, and eight (HGB, HCT, MCV, MCH, PLT, PCT, MPV, and PLCR) were significantly decreased in BB genotype mice under 8 h after LPS treatment. **(C)** A total of 18 blood parameters were significantly different at 8 h of LPS exposure compared with 0 h (without LPS treatment) in both the AA and BB genotype mice (*n* = 425; at 0 h/non-treatment: AA = 148, BB = 182; at 8 h of LPS exposure: AA = 52, BB = 43). Among these blood parameters, 8 (BASO, NEUTp, MONOp, BASOp, MCV, PDW, MPV, and PLCR) were significantly increased after 8 h LPS exposure, and 10 (WBC, LYMPH, LYMPHp, RBC, HGB, HCT, MCHC, RDW-CV, PLT, and PCT) were significantly decreased at 8 h after LPS treatment compared to 0 h (without LPS treatment). WBC, white blood cell count; NEUT, absolute neutrophil count; LYMPH, absolute lymphocyte count; MONO, absolute monocyte count; EO, absolute eosinophil count; BASO, absolute basophil count; NEUTp, neutrophil percentage; LYMPHp, lymphocyte percentage; MONOp, monocyte percentage; EOp, eosinophil percentage; BASOp, basophil percentage; RBC, red blood cell count; HGB, hemoglobin concentration; HCT, hematocrit; MCV, mean cell volume; MCH, mean corpuscular hemoglobin; MCHC, mean corpuscular hemoglobin concentration; RDW-SD, red cell distribution width-SD; RDW-CV, red cell distribution width-CV; PLT, platelet count; PCT, plateletcrit; MPV, mean platelet volume; PDW, platelet distribution width; PLCR, platelet-large cell ratio. *P < 0.05; **P < 0.01.

To explore the differences in the functions of the two haplotypes, a trait association study was first performed. Because the second mutation site of the haplotype could be detected using the restriction enzyme *Stu*I (GGCC), the genotypes of 552 Kunming mice were identified using the PCR–RFLP method. The results of the trait association analysis revealed that the A/B haplotype was significantly associated with 10 different blood parameters. Among them, the WBC, NEUT, LYMPH, and HGB were significantly increased in the AA genotype compared with the BB genotype (*P* < 0.05) (Figure 3A, Table S2 in Supplementary Material). Following LPS treatment, 14 blood parameters were significantly different between the A and B haplotypes. The mean BASO, MONO, EO, BASOp, MONOp, and EOp values were increased in BB genotype mice (*P* < 0.05). The mean values of HGB, HCT, MCV, MCH, PLT, PCT, MPV, and PLCR were significantly increased in AA genotype mice (*P* < 0.05) (Figure 3B, Table S3 in Supplementary Material). In both AA and BB genotype mice, 18 different blood parameters were significantly altered in response to LPS treatment compared with the corresponding untreated control mice. Among them, BASOp, NEUTp, MONOp, BASO, MCV, PDW, MPV, and PLCR were significantly increased in response to LPS stimulation compared with the control treatment among both AA and BB genotype mice (*P* < 0.05). In addition, the mean values of WBC, LYMPH, LYMPHp, RBC, HGB, HCT, MCHC, RDW-CV, PLT, and PCT were significantly reduced following LPS treatment in both genotypes of mice (*P* < 0.05) (Figure 3C).

### AA Genotype Kunming Mice Exhibit Stronger Inflammatory Responses than BB Genotype Kunming Mice

**Figure 4:**
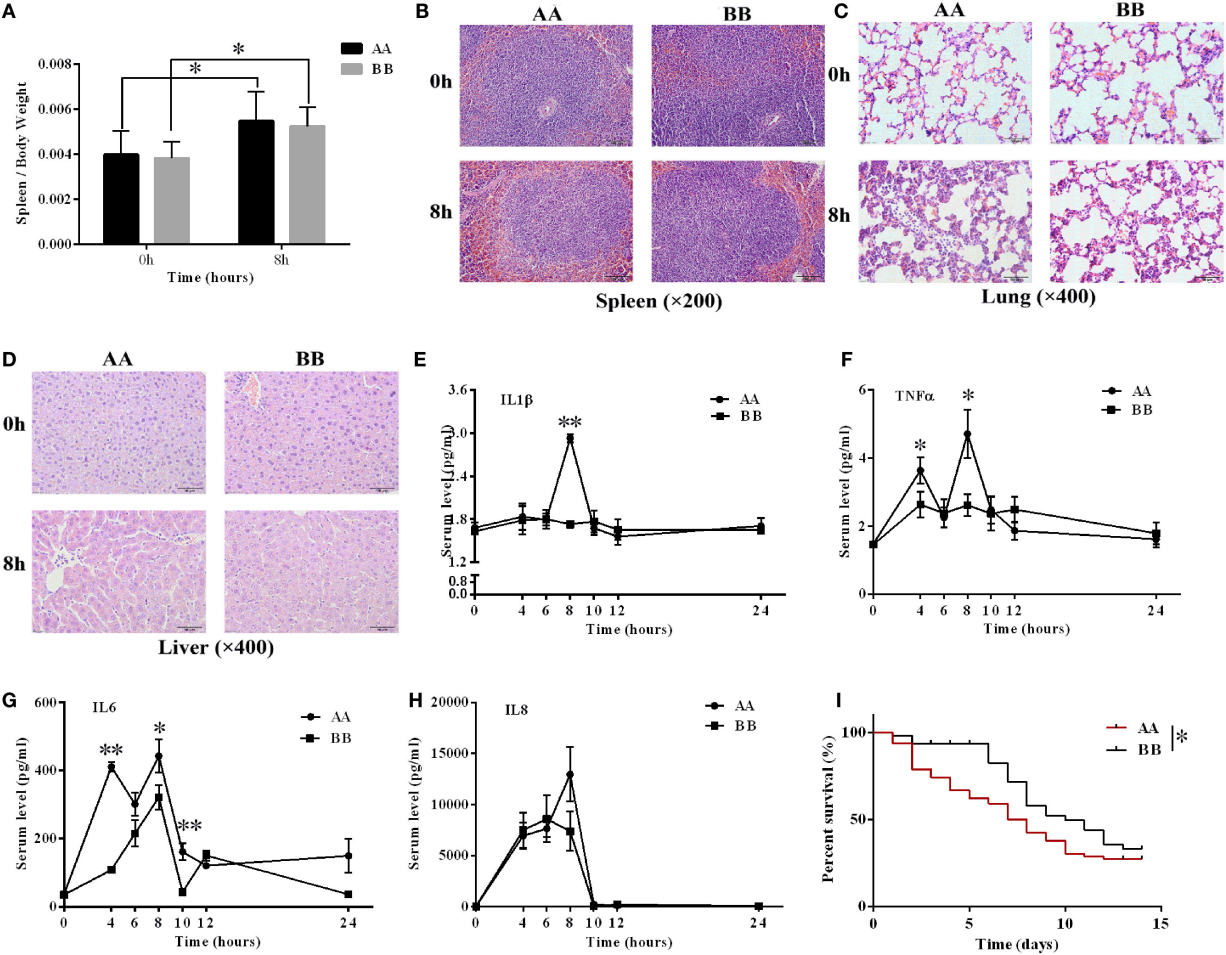
**The inflammatory response in the AA genotype Kunming mice was stronger compared with BB genotype Kunming mice**. **(A)** The weight of the spleen was significantly increased at 8 h of LPS exposure compared with non-treatment. **(B–D)** Hematoxylin and eosin (H&E) staining of spleen, lung, and liver tissues at 0 and 8 h of LPS stimulation. Spleen tissue: scale bar = 100 μm; lung and liver tissues: scale bar = 50 μm. **(E–H)** The serum *IL1*β, *TNF*α, *IL6*, and *IL8* levels were detected *via* ELISA. These cytokines were upregulated by LPS treatment, and the levels of *IL1*β, *TNF*α, and *IL6* were significantly increased in the AA genotype mice compared with the BB genotype mice (*P* < 0.05). The results are presented as the means ± SEM (*n* = 3). **(I)** The Kaplan–Meier survival curves of the AA genotype mice (*n* = 57) and the BB genotype mice (*n* = 41) were significantly different. The survival ratio of the BB genotype mice under infection of *S. typhimurium* was significantly higher than that of the AA genotype mice (*P* < 0.05). *P < 0.05; **P < 0.01.

Mice harboring the two genotypes were administered 10 mg/kg LPS *via* intraperitoneal injection. Splenomegaly symptoms were noted at 8 h after LPS injection (Figure 4A). The characteristics of the tissues were analyzed using histological analysis. Increased red pulp and reduced white pulp proportions were noted in the spleen tissue of the AA genotype mice compared with the BB genotype mice 8 h after LPS injection (Figure 4B). In the lung tissue, the alveolar wall was thicker in the AA genotype mice than in the BB genotype mice at 8 h of LPS stimulation (Figure 4C). Liver congestion and inflammatory cell infiltration symptoms were more serious in the AA genotype mice than in the BB genotype mice at 8 h of LPS stimulation (Figure 4D). We also performed TUNEL assays to compare the injury to these two genotype mice under LPS treatment. Our results showed that the proportion of apoptotic cells in the spleen and liver tissues of AA genotype mice was significantly higher than that of BB genotype mice after LPS stimulation for 8 h (Supplementary Figures 3A–C). The ELISA results revealed increased *IL1*β, *TNF*α, *IL6*, and *IL8* levels upon LPS treatment. The *IL1*β, *TNF*α, and *IL6* levels in AA genotype mice were significantly increased compared with BB genotype mice at 8 or 4 h of LPS exposure (Figures 4E–H). RNA-seq data from the spleen tissues showed that the transcriptional levels of all the detectable inflammation related cytokines were up-regulated after 4 or 8 h of LPS treatment. Moreover, the *IL1*β, *TNF*α, *IL6, IL1*α, *IL18, CCL4, CCL5*, and *CCL3* genes were relatively higher in spleen tissues from the AA genotype mice than in those from the BB genotype mice at 0 h. While the transcriptional levels of *CXCL11, IL10* and *IL12b* genes were relatively lower in spleen tissues from the AA genotype mice at 0 h. However, for all the genes, not much difference were found at mRNA level between AA and BB genotype mice at 4 or 8 h of LPS exposure (Supplementary Figure 3D). Further, the AA genotype mice exhibited significantly reduced survival time after infection with *S. typhimurium* compared with the BB genotype mice (*P* < 0.05) (Figure 4I). All of these results indicated that the inflammatory response was stronger in the AA genotype mice than in the BB genotype mice following LPS treatment or pathologic infection.

### The expression Level of *miR-155* Is Increased in AA Genotype Kunming Mice Compared With BB Genotype Kunming Mice

**Figure 5:**
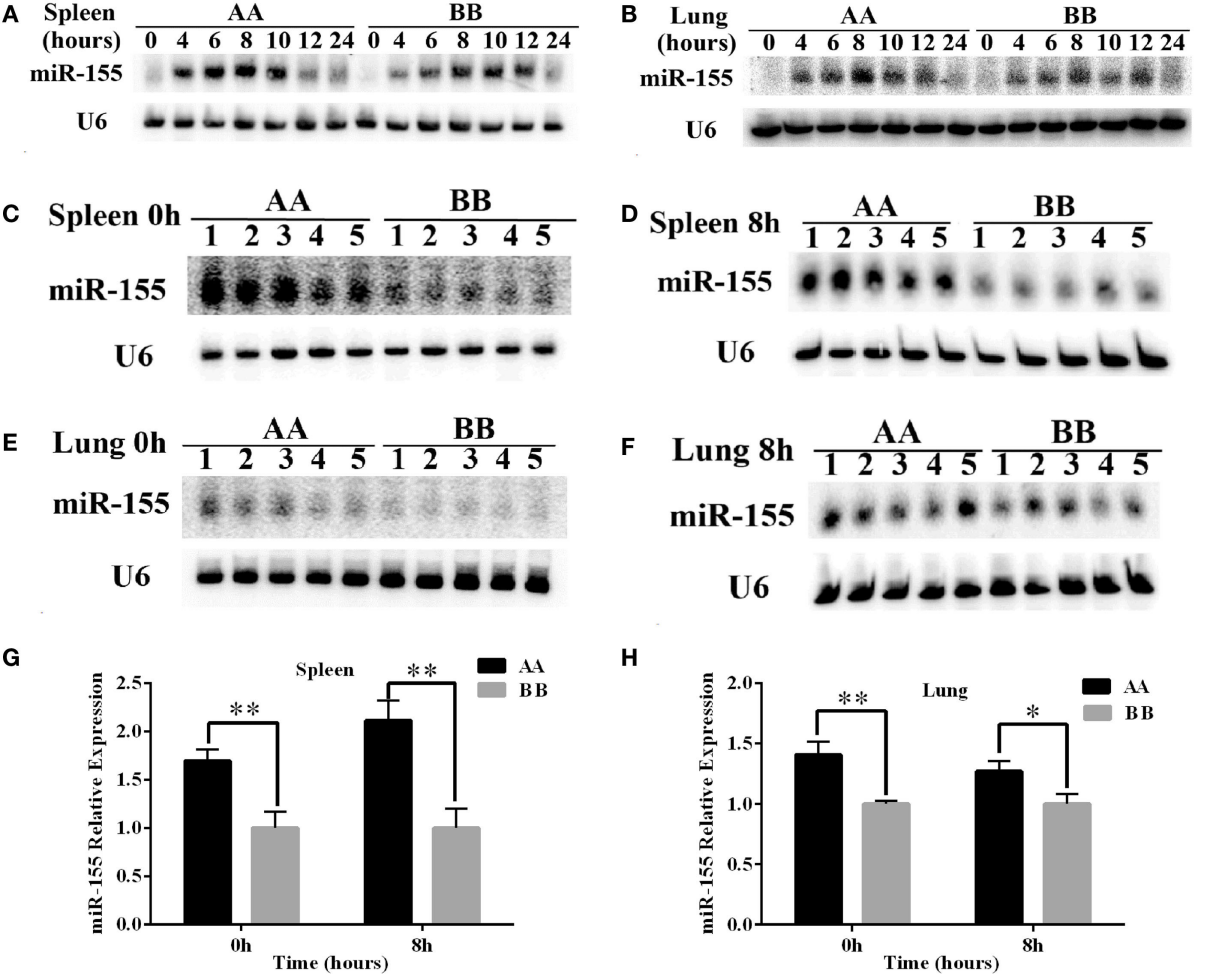
**The expression of *miR-155* was higher in AA genotype Kunming mice than in BB genotype Kunming mice**. **(A,B)** Northern blotting was used to detect the expression of *miR-155* in the spleen and lung tissues at 0, 4, 6, 8, 10, 12, and 24 h of LPS exposure. **(C–F)** Comparative analysis of the difference in *miR-155* expression in the spleen and lung tissues at 0 and 8 h of LPS stimulation between the AA and BB genotype mice. **(G,H)** Quantitative analyses of the difference in *miR-155* expression between the AA and BB genotype mice based on the northern blotting results using Quantity One software. The expression of *miR-155* was significantly increased in the AA genotype mice compared with the BB genotype mice (*P* < 0.05). The expression of *miR-155* was normalized to that of *U6*, and the results are presented as the means ± SEM (*n* = 5). *P < 0.05; **P < 0.01.

The expression levels of *miR-155* in the spleen and lung were detected by northern blotting. The results showed that *miR-155* was upregulated sharply, reaching its highest level at 8 h of LPS exposure, and then declined to approximately the normal level at 24 h of LPS exposure in the spleen and lung tissues. The trend of *miR-155* expression following LPS treatment was similar in between AA and BB genotype mice (Figures 5A,B). Moreover, *miR-155* expression in spleen tissue of AA genotype mice was approximately 1.5- to 2-fold higher than that of BB genotype mice at 0 and 8 h of LPS exposure. Additionally, *miR-155* expression in the lung tissue was significantly increased in AA genotype mice compared with BB genotype mice at 0 and 8 h of LPS exposure (Figures 5C–H). These results indicated that *miR-155* was rapidly upregulated under LPS treatment, and that AA genotype mice exhibited increased *miR-155* expression compared with BB genotype mice under both LPS treatment and non-treatment conditions.

### The Expression Levels of the Majority of *miR-155* Target Genes in the Spleen Tissue Were Reduced in AA Genotype Kunming Mice Compared With BB Genotype Kunming Mice

**Figure 6:**
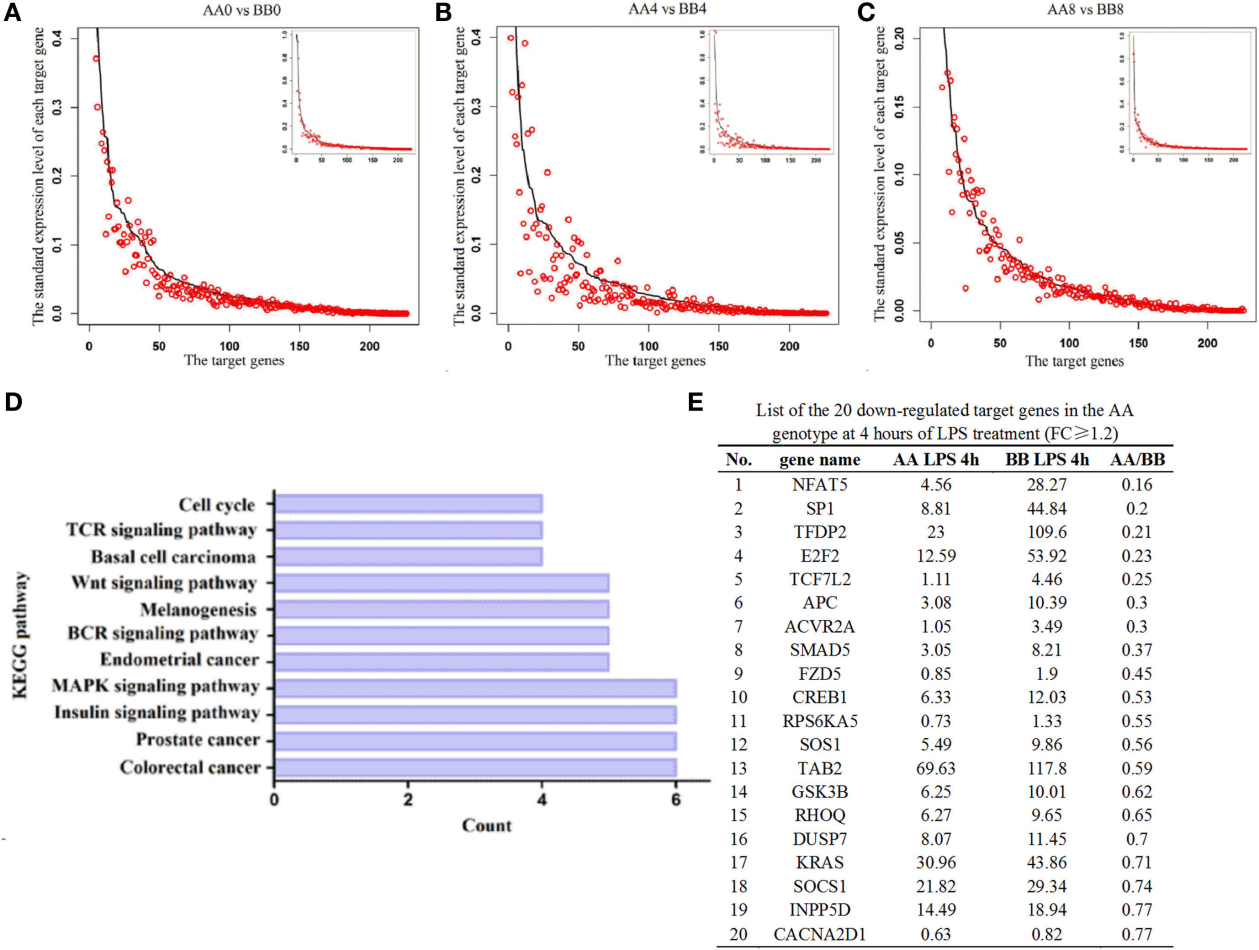
**Comparisons of the expression levels of *miR-155* target genes in spleen tissue**. **(A–C)** The dark line represents the expression levels of target genes in the BB genotype mice. The highest expression of the target gene in the BB genotype mice was normalized to one. All of the target genes were ranked from highest to lowest according to their expression levels in the BB genotype mice, and the corresponding target genes in the AA genotype mice are indicated by the red circle. The *X*-axis denotes the target gene, and the number indicates the rank of the target gene. The *Y*-axis is the standardized expression level of each target gene in the spleen tissues. The low-magnification figure contains all of the target genes, and several extremely highly expressed target genes were deleted from the high-magnification figure **(D)**. The downregulated target genes in the AA genotype at 4 h of LPS exposure (FC ≥ 1.2) were used in KEGG pathway analysis. The *X*-axis indicates the number of target genes involved in each pathway. The *Y*-axis indicates the 11 significantly enriched signaling pathways related to these downregulated target genes. **(E)** List of the 20 downregulated target genes in the AA genotype mice at 4 h of LPS exposure (FC ≥ 1.2).

To further analyze the difference in *miR-155* expression between the AA and BB genotypes, we analyzed the expression profiles of its target genes. Equal volumes of RNA from three individuals harboring each genotype who were exposed to LPS for 0, 4, or 8 h were pooled together, and the expression profiles of *miR-155* target genes were predicted using TargetScan software and were determined using RNA-seq. The results showed that 76.99% (174 genes), 73.01% (165 genes), and 58.85% (133 genes) of the target genes were downregulated in AA genotype mice compared with BB genotype mice at 0, 4, and 8 h of LPS exposure, respectively (Figures 6A–C, Table S5 in Supplementary Material). These results indicated that most of the *miR-155* target genes were downregulated at 0 and 4 h of LPS exposure in the AA genotype mice compared with the BB genotype mice. We further analyzed the signaling pathways regulated by the 20 downregulated *miR-155* target genes [fold-change (FC) ≥ 1.2] at 4 h of LPS exposure using the Kyoto Encyclopedia of Genes and Genomes (KEGG) database (Figure 6E). The results showed that the *TCR, BCR, MAPK, insulin*, and *Wn*t pathways as well as various cancer signaling pathways were targeted by *miR-155* (Figure 6D, Table S4 in Supplementary Material).

### Protein Levels of Two Important *miR-155* Target Genes Significantly Differed between the Two Genotypes of Kunming Mice

**Figure 7:**
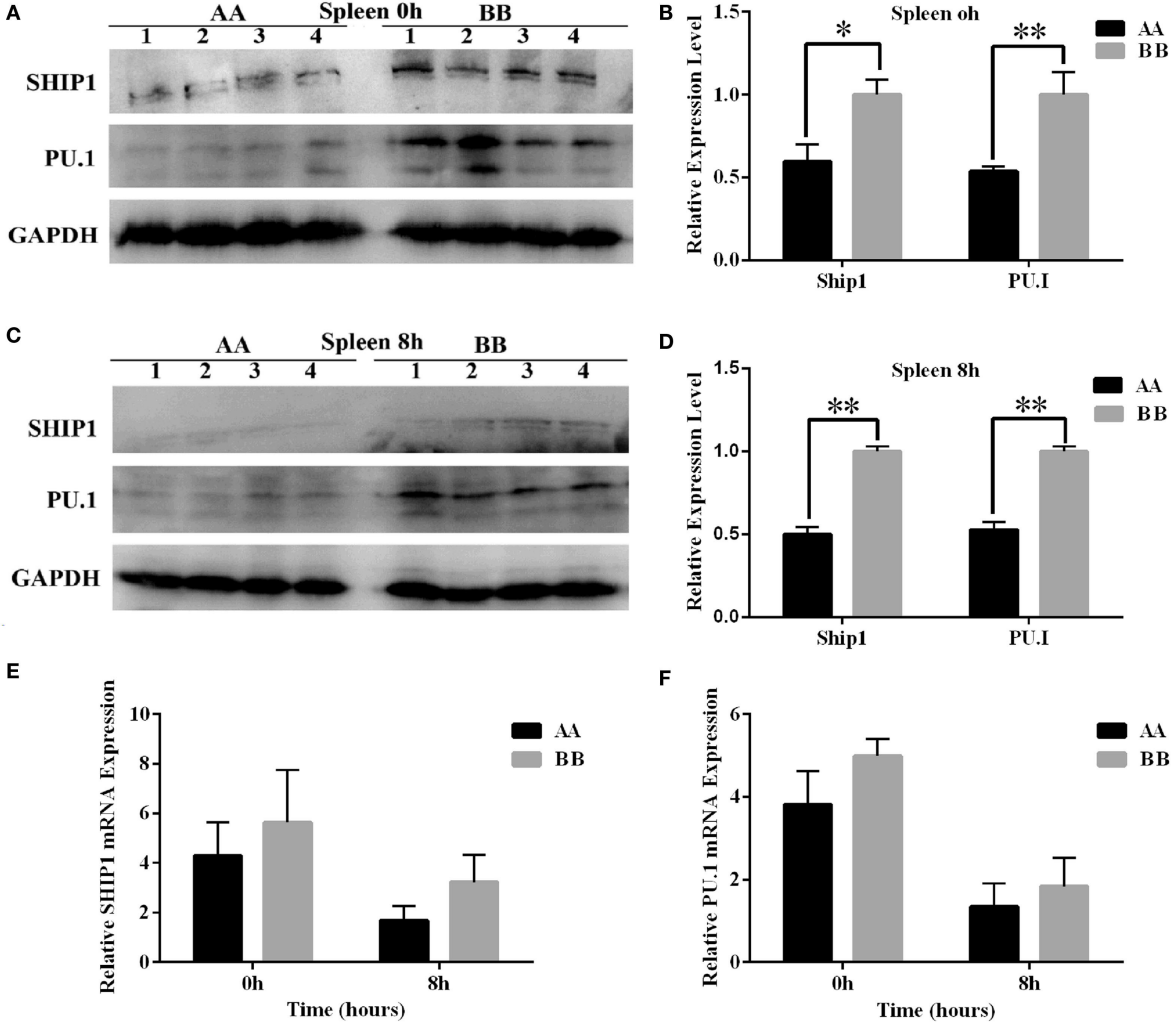
**Comparative analysis of the protein expression of *miR-155* target genes in Kunming mice**. **(A,C)** PU.1 and SHIP1 protein levels in the spleen tissues of mice at 0 and 8 h of LPS exposure were determined by western blotting. **(B,D)** Quantitative analysis of differential expression based on the western blotting results was performed using ImageJ software. **(E,F)** The mRNA levels of PU.1 and SHIP1 were detected using Q-PCR. *Tubulin* was used as an internal control. The results are presented as the means ± SEM (*n* = 4). *P < 0.05; **P < 0.01.

To further confirm the differential expression of *miR-155* between the two genotypes of mice, we measured the protein expression of two *miR-155* target genes, SHIP1 and PU.1, which are closely related to hematopoiesis. The protein levels of these two genes in the spleen tissue were significantly reduced in AA genotype mice compared with BB genotype mice at 0 and 8 h of LPS exposure (*P* < 0.05) (Figures 7A–D). Additionally, the mRNA levels of the *SHIP1* and *PU.1* genes were detected using Q-PCR. The results revealed a non-significant pattern of downregulated expression of these two genes in AA genotype mice compared with BB genotype mice (Figures 7E,F).

### The Expression Levels and Functions of *miR-155* Varied According to Its SNPs

**Figure 8:**
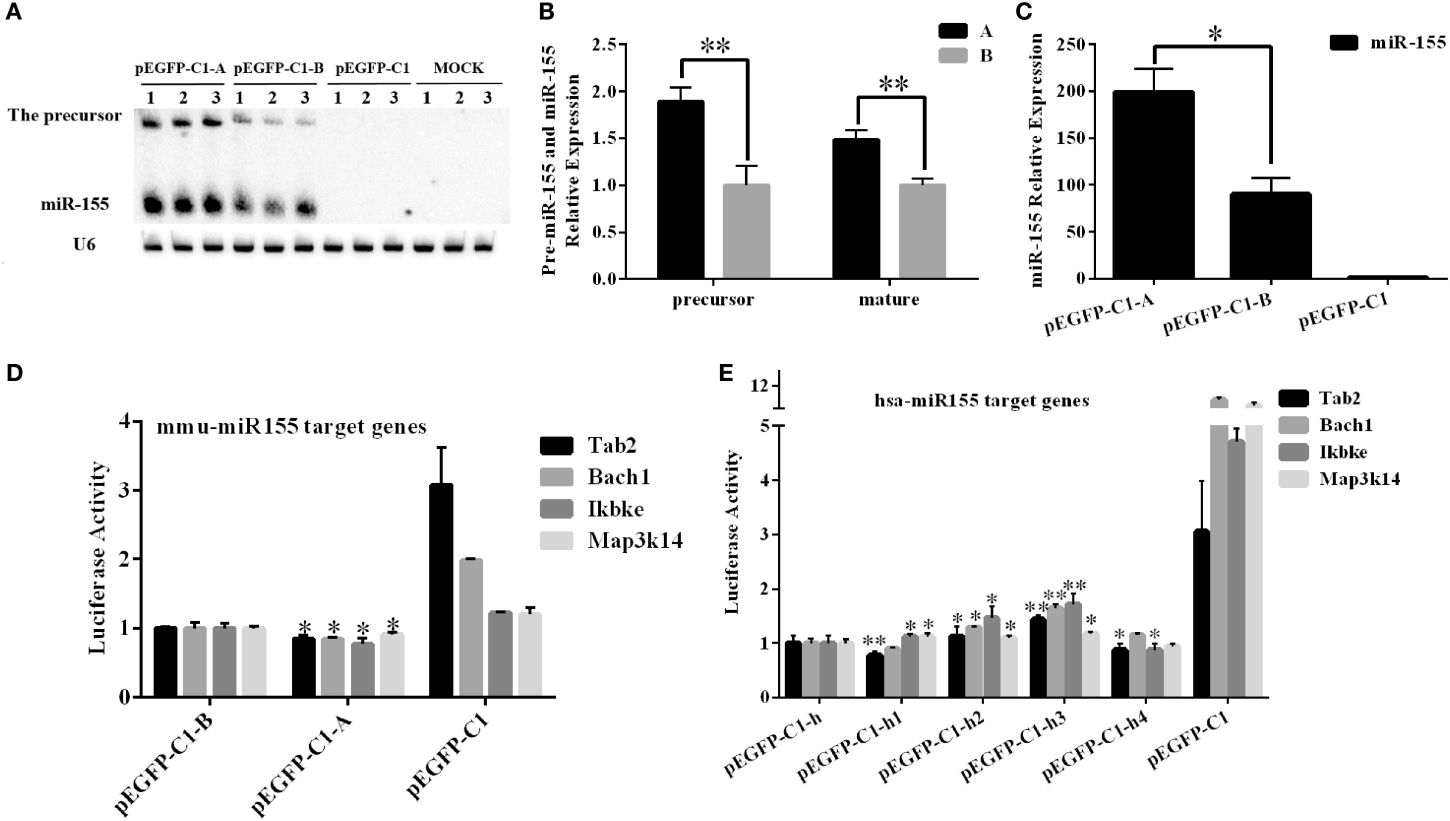
**Examination of the effects of different SNPs in mouse and human *miR-155 in vitro***. **(A,B)** A 256-bp DNA fragment containing the entire mice pre-*miR-155* sequence of the A or B haplotype was inserted into the pEGFP-C1 vector. The pEGFP-C1-A and pEGFP-C1-B constructs were then transfected into BHK-21 cells, and the expression of *miR-155* was analyzed after 24 h. **(C)** Real-time PCR was performed to detect the expression of *miR-155* after transfection of the pEGFP-C1-A/B construct. **(D)** A luciferase assay was used to analyze the expression of *miR-155* in BHK-21 cells transfected with the pEGFP-C1-A or pEGFP-C1-B construct. The pEGFP-C1-A/B construct was co-transfected into BHK-21 cells with one of the psi-check2-*mTab2/mBach1/mIkbke/mMap3k14* constructs harboring the *miR-155* binding site amplified from the 3′-untranslated region (UTR) of these four mouse genes. The luciferase activity was analyzed 24 h after transfection. The pEGFP-C1 empty vector was used as a negative control, and the luciferase activity of the pEGFP-C1-B-transfected cells was set to one. **(E)** A luciferase assay was used to analyze the functions of the human *miR-155* gene in BHK-21 cells. A 255-bp DNA fragment containing human pre-*miR-155*, including the four SNPs of interest, was inserted into the pEGFP-C1 vector. One of the pEGFP-C1-h/h1/h2/h3/h4 constructs was co-transfected into BHK-21 cells with one of the psi-check2-*hTab2/hBach1/hIkbke/hMap3k14* constructs harboring the *miR-155* binding site amplified from the 3′-untranslated region (UTR) of these four human genes. The luciferase activity was analyzed 24 h after transfection. The pEGFP-C1 empty vector was used as a negative control, and the luciferase activity of the pEGFP-C1-h-transfected cells was set to one. *U6* was used as an internal control for the expression of the mouse *miR-155* gene. The results are presented as the means ± SEM (*n* = 3). *P < 0.05; **P < 0.01.

To further confirm the differential expression of *miR-155* between the A and B haplotypes in mice, we cloned the 256-bp DNA fragment that contained the entire pre-*miR-155* sequence into the pEGFP-C1 vector. The constructed A and B haplotype vectors were labeled pEGFP-C1-A and pEGFP-C1-B, respectively. The two constructs were transfected into BHK-21 cells at equal plasmid DNA concentrations. Then, the expression levels of pre-*miR-155* and mature *miR-155* were detected by northern blotting. The expression of pre-*miR-155* and mature *miR-155* from the A haplotype construct was significantly higher by approximately 1.5-fold than their expression from the B haplotype construct (*P* < 0.05) (Figures 8A,B). Moreover, the Q-PCR results confirmed that *miR-155* expression from the A haplotype construct was significantly higher than that from the B haplotype construct (*P* < 0.05) (Figure 8C). In addition, the dual-luciferase assay results indicated that both the A and B haplotype constructs inhibited the expression of the *miR-155* target genes *Tab2, Bach1, Ikbke*, and *Map3k14*. Moreover, the A haplotype construct had a significantly stronger effect than the B haplotype construct (*P* < 0.05) (Figure 8D). *In vitro* experiments indicated that the A haplotype construct generated more mature *miR-155* than the B haplotype construct.

The 255-bp human DNA fragments, including the SNP sites of *miR-155*, were inserted into the pEGFP-C1 vector; the resulting constructs were labeled as pEGFP-C1-h/h1/h2/h3/h4. The dual-luciferase assay results indicated that the pEGFP-C1-h/h1/h2/h3/h4 constructs inhibited the expression of the human *miR-155* target genes *Tab2, Bach1, Ikbke*, and *Map3k14*. Notably, the pEGFP-C1-h2 and pEGFP-C1-h3 constructs had significantly weaker effects on these four *miR-155* target genes than pEGFP-C1-h (*P* < 0.05). However, the other two constructs did not have clear effects on the expression of these four *miR-155* target genes (Figure 8E).

### Two SNPs in the *miR-155* Haplotype Affected *miR-155* Expression in Mice

**Figure 9:**
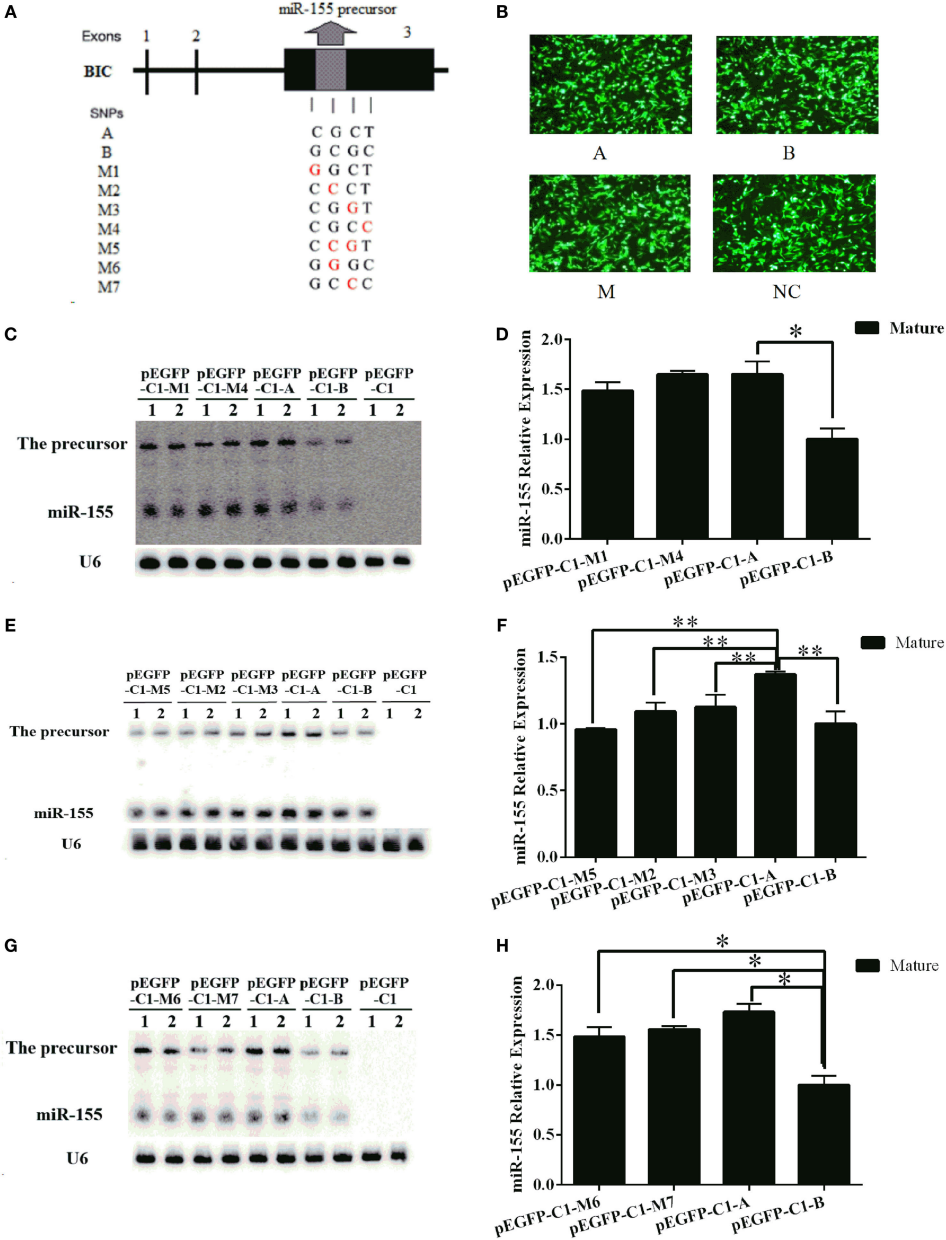
**Two important SNPs in the haplotype can affect the expression of *miR-155* in mice**. **(A)** A schematic diagram of the mouse *miR-155* gene SNPs. **(B)** BHK-21 cells were transfected with the pEGFP-C1-A/B/M1–M7 or pEGFP-C1 vector. **(C,E,G)** The expression of the mouse *miR-155* gene from each of the pEGFP-C1-A/B/M1/M2/M3/M4/M5/M6/M7 constructs was detected by northern blotting. **(D,F,H)** Quantitative analysis of the expression levels of the mouse *miR-155* gene based on northern blotting results was performed using Quantity One software. The pEGFP-C1 vector was used as a control. *U6* was used as an internal control for the expression of the mouse *miR-155* gene. The results are presented as the means ± SEM (*n* = 4). *P < 0.05; **P < 0.01.

To further explore whether functional SNPs in the haplotype contributed to the different expression levels of the mouse *miR-155* gene, we designed a series of mutant constructs (M1–M7). In these constructs, SNPs in the A haplotype were converted to the corresponding nucleotide in the *B* haplotype individually in constructs M1–M4. In M5, the two middle SNPs of the A haplotype were converted to the corresponding nucleotide in the B haplotype synchronously. In M6 and M7, the two middle SNPs of the B haplotype were individually converted to the corresponding nucleotide in A haplotype (Figure 9A). All of the mutant and normal A and B haplotype constructs were transfected into BHK-21 cells at equal plasmid DNA amounts (Figure 9B). The expression level of *miR-155* was detected by northern blotting 24 h after transfection. The expression of *miR-155* from the M1 and M4 constructs was similar to that from the A haplotype construct and was significantly increased compared with that from the B haplotype construct (Figures 9C,D). The expression of *miR-155* from the M2, M3, and M5 constructs was significantly reduced compared with that from the A haplotype construct (Figures 9E,F). The expression of *miR-155* from the M6 and M7 constructs was significantly increased compared with that from the B haplotype construct but was slightly reduced compared with that from the A haplotype construct (Figures 9G,H). The results of this point mutation analysis confirmed that the two middle SNPs contributed to the differential expression of the mouse *miR-155* gene between the A and B haplotypes. The expression level of the mouse *miR-155* gene decreased when these two SNPs of the A haplotype were converted to the corresponding nucleotide in the B haplotype and increased when these two SNPs of the B haplotype were converted to the corresponding nucleotide in the A haplotype.

## Discussion

Previous studies confirmed that *miR-155* plays crucial roles in the immune response (22, 23). However, few studies have focused on its genetic variations. Notably, the *miR-155* gene contains abundant SNPs. In this study, we identified four SNPs in the 256-bp DNA fragment containing the *miR-155* gene in mice. In fact, we detected 16 SNPs in a 1.2-kb DNA fragment in the Kunming and C57BL/6 mouse strains (data not shown). Additionally, four SNPs were identified in the human pre-*miR-155* based on sequencing data for 1,000 human genomes. In total, 720 SNPs were found in the 15-kb human genomic DNA fragment containing *miR-155* (18, 19). The high mutation rates of *miR-155* remind us of classic immune-related *MHC* genes. Previous studies reported that there were 86 SNPs per kb in the *HLA-B* gene (24, 25). The high mutation rates of *MHCs* may be beneficial for the adaptive immunity of the host (26–28). Thus, we hypothesized that a high mutation rate of *miR-155* may also be beneficial for the adaptive immunity of the host.

In mice, the four SNPs were closely linked and formed two haplotypes. The *miR-155* levels were significantly different between these two haplotypes. Two SNPs, one located in the stem–loop and the other located near the 3′ end of pre-*miR-155*, were confirmed to be responsible for the differential expression of *miR-155*. Similarly, the middle two SNPs, especially the third SNP, weakened the effects of human *miR-155* gene on all the tested target genes. We deduced that the middle two SNPs decreased the expression of mature *miR-155* in humans. Bioinformatics analysis results showed that the RNA secondary structures of the Dicer/Drosha digestion sites of in pre-*miR-155* were affected by these two SNPs in mice (Figure 1B). It is well known that mature miRNAs are processed by the RNA endonucleases Dicer and Drosha (29). Thus, we deduced that these two SNPs could affect the expression of *miR-155* by interfering with RNA digestion by Dicer or Drosha in mice. Previous studies also indicated that the expression level of miRNA could be influenced by SNPs located at Dicer/Drosha binding sites (30–32). The structure of human pre-*miR-155* was changed by the third SNP according to the RNAfold results. Furthermore, the second SNP affected the function of the human *miR-155* gene, although no evident structural variation was observed based on RNAfold analysis (Figure 2B). We deduced that the second SNP may have minor effects on the miRNA structure compared to the third SNP in humans. Correspondingly, the effects of the second SNP on the human *miR-155* gene were weaker than the effects of the third SNP based on the dual-luciferase assay. Therefore, the SNPs, that cause variation in the secondary structures of pre-miRNA, may be important for the expression and functions of the corresponding miRNAs.

The expression of *miR-155* target genes differed between the two genotypes under normal and LPS stimulation conditions in mice. These results were consistent with the differential expression of *miR-155* between the two genotypes. Moreover, we found in both genotypes that the differences in the expression of *miR-155* target genes peaked at 4 h of LPS exposure, and that these differences nearly completely disappeared at 8 h of LPS exposure. One possible reason for this result may be that *miR-155* expression reaches a very high level in both genotypes at 8 h of LPS exposure. Therefore, the genotype effects on the differential expression of *miR-155* were reduced. In addition, pro-inflammatory and anti-inflammatory factors regulate the balance of the inflammatory response (33–35). After a relatively long period of LPS exposure, many pro-inflammatory and anti-inflammatory factors may intervene in the expression of *miR-155* target genes, which could also weaken the genotype effect on the differential expression of *miR-155*. In addition, we found that mice harboring the AA genotype of *miR-155* have stronger inflammatory response under LPS treatment. The protein levels of inflammatory factors were significantly higher in AA genotype mice after LPS treatment *via* ELISA detection. Whereas, the mRNA level after LPS treatment did not show much difference between AA and BB genotype mice according to the RNA-seq data. We deduced the main reason was that the genes reached to the maximum transcriptional levels in AA genotype mice earlier than that in BB genotype mice, although the maximum level were almost same in these two genotype mice. Therefore, they could accumulate more proteins in AA genotype mice than in BB genotype mice.

*miR-155* participates in the regulation of the development of many specific T lymphocyte subsets (2, 8, 36, 37). Few studies have focused on the regulation of blood parameters by *miR-155*. In *Sus scrofa (pig)*, one SNP of *miR-155* was associated with blood parameters (38). In this study, we also found that *miR-155* was associated with various blood parameters in mice. No difference was found in body weight between the two genotypes. This finding indicated that the differences in *miR-155* and blood parameters were not influenced by body growth under normal conditions. However, the survival time was significantly different between the two mouse genotypes upon infection with *S. typhimurium*. These results indicated that the different expression levels of *miR-155* were not sufficient to affect growth under normal conditions but could cause observable phenotypic differences under pathological conditions.

Moreover, *miR-155* expression was rapidly altered by LPS stimulation. *miR-155* was observably upregulated at 4 h of LPS exposure. Previous studies also found that *miR-155* is rapidly upregulated by LPS, poly(I:C), or IFNβ treatment (17, 39, 40). Furthermore, we found that *miR-155* expression began to decline at approximately 10 h of LPS exposure and had returned to normal levels at 24 h of LPS exposure. These results indicated that *miR-155* degraded rapidly in the host. Other studies demonstrated that upregulation of *miR-155* could persist for greater than 24 h after LPS treatment in RAW 264.7 macrophages (41, 42); that finding was not consistent with our results. One possible reason for that finding may be that responses by RAW 264.7 macrophages do not mimic *in vivo* responses, especially regarding the degradation of miRNA. Thus, we concluded that *miR-155* acts during the acute phase of the immune response. Furthermore, according to our signaling pathway analysis, *miR-155* was involved in the *TCR* and *BCR* signaling pathways. The roles of *miR-155* in these signaling pathways were confirmed in previous studies (4, 36, 40). We also found that *miR-155* was involved in the *MAPK, insulin*, and *Wnt* signaling pathways. One previous study indicated that *miR-155* was upregulated *via* the *MAPK/NF*κ*B* signaling pathway in RAW 264.7 macrophages in response to stimulation with adiponectin (43). These results indicated that *miR-155* can participate in the immune response by targeting numerous pathways.

Additionally, some studies indicated that certain mutations of *miR-155* were associated with trisomy 21 (44), eczema (45), and multiple sclerosis (46) in clinic. In our study, we found specific functional SNPs in human *miR-155*. Therefore, the mutations reported in this study may also relate to these diseases.

In conclusion, we identified two natural functional SNPs of *miR-155* in both humans and mice. The *miR-155* expression levels, blood parameters, and inflammatory responses differed between mice harboring different haplotypes. Moreover, we confirmed that two important SNPs in the haplotypes were responsible for the differential expression of *miR-155* in mice. Moreover, these two SNPs affected the functions of human *miR-155*. Our study provides the first evidence that natural *miR-155* SNPs can affect its expression and the host immune response.

## Author Contributions

SZ and XL conceived and designed the study. CL, HH (author #2), SH, and HW performed the *in vitro* experiments. CL, CZ, and JN performed the *in vivo* experiments. CL, HH (author #5), and HL processed the tissue sections. AL, LJ, LY, and XL analyzed the RNA-seq data. CL, XL, and SZ wrote the manuscript. All authors read and approved the final manuscript.

## Conflict of Interest Statement

The authors declare that the research was conducted in the absence of any commercial or financial relationships that could be construed as a potential conflict of interest. The reviewer KL and handling Editor declared their shared affiliation, and the handling Editor states that the process nevertheless met the standards of a fair and objective review.

## References

[1] Sun G Yan J Noltner K Feng J Li H Sarkis DA SNPs in human miRNA genes affect biogenesis and function. RNA (2009) 15:1640–51. 10.1261/rna.1560209 19617315 PMC2743066

[2] Gracias DT Stelekati E Hope JL Boesteanu AC Doering TA Norton J The microRNA miR-155 controls CD8(+) T cell responses by regulating interferon signaling. Nat Immunol (2013) 14:593–602. 10.1038/ni.2576 23603793 PMC3664306

[3] Georgantas RW III Hildreth R Morisot S Alder J Liu CG Heimfeld S CD34+ hematopoietic stem-progenitor cell microRNA expression and function: a circuit diagram of differentiation control. Proc Natl Acad Sci U S A (2007) 104:2750–5. 10.1073/pnas.0610983104 17293455 PMC1796783

[4] Rodriguez A Vigorito E Clare S Warren MV Couttet P Soond DR Requirement of bic/microRNA-155 for normal immune function. Science (2007) 316:608–11. 10.1126/science.1139253 17463290 PMC2610435

[5] Thai TH Calado DP Casola S Ansel KM Xiao C Xue Y Regulation of the germinal center response by microRNA-155. Science (2007) 316:604–8. 10.1126/science.1141229 17463289

[6] O’Connell RM Kahn D Gibson WS Round JL Scholz RL Chaudhuri AA MicroRNA-155 promotes autoimmune inflammation by enhancing inflammatory T cell development. Immunity (2010) 33:607–19. 10.1016/j.immuni.2010.09.009 20888269 PMC2966521

[7] Kohlhaas S Garden OA Scudamore C Turner M Okkenhaug K Vigorito E. Cutting edge: the Foxp3 target miR-155 contributes to the development of regulatory T cells. J Immunol (2009) 182:2578–82. 10.4049/jimmunol.0803162 19234151

[8] Lind EF Ohashi PS. Mir-155, a central modulator of T-cell responses. Eur J Immunol (2014) 44:11–5. 10.1002/eji.201343962 24571026

[9] O’Connell RM Rao DS Chaudhuri AA Boldin MP Taganov KD Nicoll J Sustained expression of microRNA-155 in hematopoietic stem cells causes a myeloproliferative disorder. J Exp Med (2008) 205:585–94. 10.1084/jem.20072108 18299402 PMC2275382

[10] O’Connell RM Chaudhuri AA Rao DS Baltimore D. Inositol phosphatase SHIP1 is a primary target of miR-155. Proc Natl Acad Sci U S A (2009) 106:7113–8. 10.1073/pnas.0902636106 19359473 PMC2678424

[11] Vigorito E Perks KL Abreu-Goodger C Bunting S Xiang Z Kohlhaas S microRNA-155 regulates the generation of immunoglobulin class-switched plasma cells. Immunity (2007) 27:847–59. 10.1016/j.immuni.2007.10.009 18055230 PMC4135426

[12] Imaizumi T Tanaka H Tajima A Yokono Y Matsumiya T Yoshida H IFN-gamma and TNF-alpha synergistically induce microRNA-155 which regulates TAB2/IP-10 expression in human mesangial cells. Am J Nephrol (2010) 32:462–8. 10.1159/000321365 20948191

[13] Gottwein E Mukherjee N Sachse C Frenzel C Majoros WH Chi JT A viral microRNA functions as an orthologue of cellular miR-155. Nature (2007) 450:1096–9. 10.1038/nature05992 18075594 PMC2614920

[14] Institute of Laboratory Animal Research, Commission on Life Sciences, National Reasearch Council. Guide for the Care and Use of Laboratory Animals. Washington, DC: National Academy Press (1996). 140 p.

[15] Hirsch E Irikura VM Paul SM Hirsh D. Functions of interleukin 1 receptor antagonist in gene knockout and overproducing mice. Proc Natl Acad Sci U S A (1996) 93:11008–13. 10.1073/pnas.93.20.11008 8855299 PMC38274

[16] Li L Bhatia M Zhu YZ Zhu YC Ramnath RD Wang ZJ Hydrogen sulfide is a novel mediator of lipopolysaccharide-induced inflammation in the mouse. FASEB J (2005) 19:1196–8. 10.1096/fj.04-3583fje 15863703

[17] Guo ZL Ren T Xu L Zhang L Yin Q Wang JC The microRNAs expression changes rapidly in mice lung tissue during lipopolysaccharide-induced acute lung injury. Chin Med J (2013) 126:181–3. 10.3760/cma.j.issn.0366-6999.20112744 23286498

[18] Genomes Project C Auton A Brooks LD Durbin RM Garrison EP Kang HM A global reference for human genetic variation. Nature (2015) 526:68–74. 10.1038/nature15393 26432245 PMC4750478

[19] Sudmant PH Rausch T Gardner EJ Handsaker RE Abyzov A Huddleston J An integrated map of structural variation in 2,504 human genomes. Nature (2015) 526:75–81. 10.1038/nature15394 26432246 PMC4617611

[20] Trapnell C Roberts A Goff L Pertea G Kim D Kelley DR Differential gene and transcript expression analysis of RNA-seq experiments with TopHat and Cufflinks. Nat Protoc (2012) 7:562–78. 10.1038/nprot.2012.016 22383036 PMC3334321

[21] Chen C Ridzon DA Broomer AJ Zhou Z Lee DH Nguyen JT Real-time quantification of microRNAs by stem-loop RT-PCR. Nucleic Acids Res (2005) 33:e179. 10.1093/nar/gni178 16314309 PMC1292995

[22] Vigorito E Kohlhaas S Lu D Leyland R. miR-155: an ancient regulator of the immune system. Immunol Rev (2013) 253:146–57. 10.1111/imr.12057 23550644

[23] Faraoni I Antonetti FR Cardone J Bonmassar E. miR-155 gene: a typical multifunctional microRNA. Biochim Biophys Acta (2009) 1792:497–505. 10.1016/j.bbadis.2009.02.013 19268705

[24] Mungall AJ Palmer SA Sims SK Edwards CA Ashurst JL Wilming L The DNA sequence and analysis of human chromosome 6. Nature (2003) 425:805–11. 10.1038/nature02055 14574404

[25] Horton R Wilming L Rand V Lovering RC Bruford EA Khodiyar VK Gene map of the extended human MHC. Nat Rev Genet (2004) 5:889–99. 10.1038/nrg1489 15573121

[26] Potts WK Slev PR. Pathogen-based models favoring MHC genetic diversity. Immunol Rev (1995) 143:181–97. 10.1111/j.1600-065X.1995.tb00675.x 7558076

[27] Messaoudi I Guevara Patino JA Dyall R LeMaoult J Nikolich-Zugich J. Direct link between mhc polymorphism, T cell avidity, and diversity in immune defense. Science (2002) 298:1797–800. 10.1126/science.1076064 12459592

[28] Sommer S. The importance of immune gene variability (MHC) in evolutionary ecology and conservation. Front Zool (2005) 2:16. 10.1186/1742-9994-2-16 16242022 PMC1282567

[29] Kuehbacher A Urbich C Zeiher AM Dimmeler S. Role of Dicer and Drosha for endothelial microRNA expression and angiogenesis. Circ Res (2007) 101:59–68. 10.1161/CIRCRESAHA.107.153916 17540974

[30] Duan R Pak C Jin P. Single nucleotide polymorphism associated with mature miR-125a alters the processing of pri-miRNA. Hum Mol Genet (2007) 16:1124–31. 10.1093/hmg/ddm062 17400653

[31] Hu Z Chen J Tian T Zhou X Gu H Xu L Genetic variants of miRNA sequences and non-small cell lung cancer survival. J Clin Invest (2008) 118:2600–8. 10.1172/JCI34934 18521189 PMC2402113

[32] Qi L Hu Y Zhan Y Wang J Wang BB Xia H-F A SNP site in pri-miR-124 changes mature miR-124 expression but no contribution to Alzheimer’s disease in a Mongolian population. Neurosci Lett (2012) 515:1–6. 10.1016/j.neulet.2012.02.061 22430032

[33] Elenkov IJ Chrousos GP. Stress hormones, Th1/Th2 patterns, pro/anti-inflammatory cytokines and susceptibility to disease. Trends Endocrinol Metab (1999) 10:359–68. 10.1016/S1043-2760(99)00188-5 10511695

[34] Borovikova LV Ivanova S Zhang M Yang H Botchkina GI Watkins LR Vagus nerve stimulation attenuates the systemic inflammatory response to endotoxin. Nature (2000) 405:458–62. 10.1038/35013070 10839541

[35] Gideon HP Phuah J Myers AJ Bryson BD Rodgers MA Coleman MT Variability in tuberculosis granuloma T cell responses exists, but a balance of pro- and anti-inflammatory cytokines is associated with sterilization. PLoS Pathog (2015) 11:e1004603. 10.1371/journal.ppat.1004603 25611466 PMC4303275

[36] Dudda JC Salaun B Ji Y Palmer DC Monnot GC Merck E MicroRNA-155 is required for effector CD8+ T cell responses to virus infection and cancer. Immunity (2013) 38:742–53. 10.1016/j.immuni.2012.12.006 23601686 PMC3788592

[37] Spoerl D Duroux-Richard I Louis-Plence P Jorgensen C. The role of miR-155 in regulatory T cells and rheumatoid arthritis. Clin Immunol (2013) 148:56–65. 10.1016/j.clim.2013.03.010 23649045

[38] Li C He H Zhu M Zhao S Li X. Molecular characterisation of porcine miR-155 and its regulatory roles in the TLR3/TLR4 pathways. Dev Comp Immunol (2013) 39:110–6. 10.1016/j.dci.2012.01.001 22301067

[39] Tili E Michaille JJ Cimino A Costinean S Dumitru CD Adair B Modulation of miR-155 and miR-125b levels following lipopolysaccharide/TNF-alpha stimulation and their possible roles in regulating the response to endotoxin shock. J Immunol (2007) 179:5082–9. 10.4049/jimmunol.179.8.5082 17911593

[40] O’Connell RM Taganov KD Boldin MP Cheng G Baltimore D. MicroRNA-155 is induced during the macrophage inflammatory response. Proc Natl Acad Sci USA (2007) 104:1604–9. 10.1073/pnas.0610731104 17242365 PMC1780072

[41] Ruggiero T Trabucchi M De Santa F Zupo S Harfe BD McManus MT LPS induces KH-type splicing regulatory protein-dependent processing of microRNA-155 precursors in macrophages. FASEB J (2009) 23:2898–908. 10.1096/fj.09-131342 19423639

[42] Richmond TK Tili E Chiabai M Palmieri D Brown M Croce C Functional interaction of mir-155, a pro-inflammatory microRNA, and quaking in the innate immune response. J Allergy Clin Immunol (2015) 135:AB97 10.1016/j.jaci.2014.12.1252

[43] Subedi A Park PH Autocrine and paracrine modulation of microRNA-155 expression by globular adiponectin in RAW 264.7 macrophages: involvement of MAPK/NF-kappaB pathway. Cytokine (2013) 64:638–41. 10.1016/j.cyto.2013.09.011 24084329

[44] Sethupathy P Borel C Gagnebin M Grant GR Deutsch S Elton TS Human microRNA-155 on chromosome 21 differentially interacts with its polymorphic target in the AGTR1 3′ untranslated region: a mechanism for functional single-nucleotide polymorphisms related to phenotypes. Am J Hum Genet (2007) 81:405–13. 10.1086/519979 17668390 PMC1950808

[45] Saaf A Kockum I Wahlgren CF Xu N Sonkoly E Stahle M Are BIC (miR-155) polymorphisms associated with eczema susceptibility? Acta Derm Venereol (2013) 93:366–7. 10.2340/00015555-1466 23047434

[46] Paraboschi EM Solda G Gemmati D Orioli E Zeri G Benedetti MD Genetic association and altered gene expression of mir-155 in multiple sclerosis patients. Int J Mol Sci (2011) 12:8695–712. 10.3390/ijms12128695 22272099 PMC3257096

