# Supplementary material for: Natural Functional SNPs in miR-155 Alter Its Expression Level, Blood Cell Counts, and Immune Responses
Source: Front Immunol. 2016 Aug 2;7:295. doi: 10.3389/fimmu.2016.00295 (PMC4970381; doi:10.3389/fimmu.2016.00295)
Supplement: Supplementary file 1 [file table_1.doc]

| **Supplementary Table S1.** **Sequences of the Q-PCR primers and the northern blot probes.** | |
| --- | --- |
|  |  |
| Primer Name | Sequence (5' to 3') |
| RT-loop-mmu-miR-155 | CTCAACTGGTGTCGTGGAGTCGGCAATTCAGTTGAGACCCCTAT |
| Taq-155-F | TCGGCAGGTTAATGCTAATTGTG |
| Taq-155-R | TCAACTGGTGTCGTGGAGTCGGC |
| mmu-U6-S | GTGCTCGCTTCGGCAGCACATAT |
| mmu-U6-A | AAAATATGGAACGCTTCACGAA |
| mmu-ship-F* | GAGCGGGATGAATCCAGTGG |
| mmu-ship-R* | GGACCTCGGTTGGCAATGTA |
| mmu-pu1-F | TGGAGAAAGCCATAGCGATCA |
| mmu-pu1-R | GCACCATGGGAGTATCGAGG |
| mmu-TUB-F | GACTATGGACTCCGTTCGCTC |
| mmu-TUB-R | TATTCTTCCCGGATCTTGCTG |
| mmu-miR-155 probe | ACCCCTATCACAATTAGCATTAA |
| U6 probe | CATGCTAAATCTTCTCTGTAT |

*O'Connell et al., (2009).
