# Supplementary material for: Natural Functional SNPs in miR-155 Alter Its Expression Level, Blood Cell Counts, and Immune Responses
Source: Front Immunol. 2016 Aug 2;7:295. doi: 10.3389/fimmu.2016.00295 (PMC4970381; doi:10.3389/fimmu.2016.00295)
Supplement: Supplementary file 2 [file table_2.doc]

| **Supplementary Table S2.** **Association analysis of two genotypes of mice miR-155 with blood parameters under normal conditions.** | | | | | | | |
| --- | --- | --- | --- | --- | --- | --- | --- |
|  |  |  |  |  |  |  |  |
| Genotype | AA | AB | BB | P | P_AA-BB_ | P_AA-AB_ | P_AB-BB_ |
| N | 148 | 222 | 182 |  |  |  |  |
| weight (g) | 26.38±0.33 | 27.03±0.27 | 26.68±0.30 | ns | ns | ns | ns |
| WBC (10^9/L) | 4.47±0.10 | 4.38±0.08 | 4.08±0.09 | <0.05 | <0.01 | ns | <0.05 |
| BASOp (‰) | 0.59±0.01 | 0.54±0.01 | 0.45±0.01 | ns | ns | ns | ns |
| NEUTp (%) | 29.17±0.53 | 28.41±0.44 | 28.51±0.48 | ns | ns | ns | ns |
| LYMPHp (%) | 64.77±0.7 | 65.6±0.57 | 64.26±0.63 | ns | ns | ns | ns |
| MONOp (%) | 2.55±0.14 | 3.14±0.11 | 2.77±0.13 | <0.01 | ns | <0.01 | <0.05 |
| EOp (%) | 2.76±0.13 | 2.76±0.1 | 2.76±0.11 | ns | ns | ns | ns |
| BASO (10^6^/L) | 2.78±0.36 | 2.32±0.29 | 1.88±0.32 | ns | ns | ns | ns |
| NEUT (10^9^/L) | 1.29±0.03 | 1.23±0.03 | 1.15±0.03 | <0.05 | <0.01 | ns | ns |
| LYMPH (10^9^/L) | 2.90±0.08 | 2.88±0.06 | 2.63±0.07 | <0.05 | <0.05 | ns | <0.05 |
| MONO (10^8^/L) | 1.14±0.01 | 1.38±0.01 | 1.16±0.01 | <0.05 | ns | <0.05 | <0.05 |
| EO (10^8^/L) | 1.21±0.01 | 1.19±0.01 | 1.12±0.01 | ns | ns | ns | ns |
| RBC (10^12^/L) | 10.03±0.06 | 9.78±0.05 | 9.92±0.06 | <0.05 | ns | <0.01 | ns |
| HGB (g/L) | 148.71±0.93 | 145.36±0.76 | 146.18±0.84 | <0.05 | <0.05 | <0.01 | ns |
| HCT (CV%) | 51.82±0.29 | 50.90±0.24 | 51.11±0.26 | <0.05 | ns | <0.05 | ns |
| MCV (fL) | 51.73±0.21 | 52.17±0.17 | 51.59±0.19 | ns | ns | ns | <0.05 |
| MCH (pg) | 14.83±0.05 | 14.88±0.44 | 14.74±0.04 | ns | ns | ns | <0.05 |
| MCHC (g/L) | 286.93±0.87 | 285.65±0.71 | 286.03±0.79 | ns | ns | ns | ns |
| RDW-SD (fL) | 31.85±0.24 | 32.95±0.20 | 32.10±0.22 | <0.01 | ns | <0.01 | <0.01 |
| RDW-CV (%) | 20.41±0.11 | 20.61±0.09 | 20.51±0.10 | ns | ns | ns | ns |
| PLT (10^10^/L) | 108.10±1.96 | 105.06±1.60 | 105.82±1.76 | ns | ns | ns | ns |
| PCT (CV%) | 0.70±40.01 | 0.72±0.01 | 0.72±0.01 | ns | ns | ns | ns |
| PDW (fL) | 6.98±0.04 | 6.87±0.04 | 6.94±0.04 | ns | ns | ns | ns |
| MPV (fL) | 6.84±0.03 | 6.77±0.03 | 6.82±0.03 | ns | ns | ns | ns |
| PLCR (%) | 5.60±0.14 | 5.12±0.12 | 5.40±0.13 | <0.05 | ns | <0.05 | ns |
