# Supplementary material for: Natural Functional SNPs in miR-155 Alter Its Expression Level, Blood Cell Counts, and Immune Responses
Source: Front Immunol. 2016 Aug 2;7:295. doi: 10.3389/fimmu.2016.00295 (PMC4970381; doi:10.3389/fimmu.2016.00295)
Supplement: Supplementary file 3 [file table_3.doc]

| **Supplementary Table S3. Comparative analysis of the differences of blood parameters between two genotypes of mice miR-155 at 8 h of LPS treatment.** | | | |
| --- | --- | --- | --- |
|  |  |  |  |
| Genotype | AA | BB | p |
| N | 52 | 43 |  |
| weight (g) | 26.91±0.18 | 26.40±0.19 | ns |
| WBC (10^9^/L) | 3.67±0.26 | 3.30±0.28 | ns |
| BASOp (%) | 0.21±0.03 | 0.43±0.03 | <0.01 |
| NEUTp (%) | 38.41±3.09 | 38.70±3.33 | ns |
| LYMPHp (%) | 32.61±2.83 | 38.10±3.05 | ns |
| MONOp (%) | 3.55±0.45 | 5.50±0.48 | <0.01 |
| EOp (%) | 1.93±0.26 | 3.47±0.29 | <0.01 |
| BASO (10^6^/L) | 7.11±0.88 | 11.03±0.95 | <0.01 |
| NEUT (10^9^/L) | 1.22±0.12 | 1.13±0.13 | ns |
| LYMPH (10^9^/L) | 1.09±0.14 | 1.18±0.15 | ns |
| MONO (10^8^/L) | 1.24±0.24 | 1.96±0.26 | <0.05 |
| EO (10^8^/L) | 0.61±0.11 | 1.09±0.12 | <0.01 |
| RBC (10^12^/L) | 9.30±0.09 | 9.21±0.11 | ns |
| HGB (g/L) | 140.06±1.30 | 130.19±1.41 | <0.05 |
| HCT (CV%) | 50.06±0.42 | 48.33±0.46 | <0.01 |
| MCV (fL) | 53.84±0.33 | 52.56±0.36 | <0.05 |
| MCH (pg) | 15.04±0.07 | 14.80±0.08 | <0.05 |
| MCHC (g/L) | 279.80±1.46 | 281.90±1.57 | ns |
| RDW-SD (fL) | 31.41±0.35 | 30.43±0.38 | ns |
| RDW-CV (%) | 18.76±0.21 | 18.58±0.23 | ns |
| PLT (10^10^/L) | 72.37±2.42 | 62.70±2.61 | <0.01 |
| PCT (CV%) | 0.54±0.01 | 0.46±0.01 | <0.01 |
| PDW (fL) | 7.64±0.08 | 7.46±0.09 | ns |
| MPV (fL) | 7.54±0.05 | 7.36±0.05 | <0.05 |
| PLCR (%) | 9.12±0.30 | 7.83±0.32 | <0.01 |
