# Supplementary material for: Natural Functional SNPs in miR-155 Alter Its Expression Level, Blood Cell Counts, and Immune Responses
Source: Front Immunol. 2016 Aug 2;7:295. doi: 10.3389/fimmu.2016.00295 (PMC4970381; doi:10.3389/fimmu.2016.00295)
Supplement: Supplementary file 4 [file table_4.doc]

| **Supplementary Table S4. Eleven enriched KEGG pathways and their involved target genes of miR-155 in the spleen tissues of mice.** | | | |  |
| --- | --- | --- | --- | --- |
|  |  |  |  |  |
| **No.** | **Term** | **Count** | **Genes** | **P** |
| 1 | Colorectal cancer | 6 | KRAS, SOS1, GSK3B, FZD5, TCF7L2, APC | <0.01 |
| 2 | Prostate cancer | 6 | E2F2, KRAS, CREB1, SOS1, GSK3B, TCF7L2 | <0.01 |
| 3 | Insulin signaling pathway | 6 | KRAS, SOS1, GSK3B, SOCS1, RHOQ, INPP5D | <0.01 |
| 4 | MAPK signaling pathway | 6 | RPS6KA5, CACNA2D1, KRAS, SOS1, TAB2, DUSP7 | <0.05 |
| 5 | Endometrial cancer | 5 | KRAS, SOS1, GSK3B, TCF7L2, APC | <0.01 |
| 6 | B cell receptor signaling pathway | 5 | KRAS, SOS1, GSK3B, NFAT5, INPP5D | <0.01 |
| 7 | Melanogenesis | 5 | KRAS, CREB1, GSK3B, FZD5, TCF7L2 | <0.01 |
| 8 | Wnt signaling pathway | 5 | GSK3B, NFAT5, FZD5, TCF7L2, APC | <0.05 |
| 9 | Basal cell carcinoma | 4 | GSK3B, FZD5, TCF7L2, APC | <0.01 |
| 10 | T cell receptor signaling pathway | 4 | KRAS, SOS1, GSK3B, NFAT5 | <0.05 |
| 11 | Cell cycle | 4 | E2F2, GSK3B, TFDP2, WEE1 | <0.05 |
|  | | | | |
