# Supplementary material for: Natural Functional SNPs in miR-155 Alter Its Expression Level, Blood Cell Counts, and Immune Responses
Source: Front Immunol. 2016 Aug 2;7:295. doi: 10.3389/fimmu.2016.00295 (PMC4970381; doi:10.3389/fimmu.2016.00295)
Supplement: Supplementary file 5 [file table_5.doc]

| **Supplementary Table S5. The expression levels of miR-155 target genes in the spleen tissues of mice through RNA-seq analysis.** | | | | | | |
| --- | --- | --- | --- | --- | --- | --- |
|  |  |  |  |  |  |  |
| Gene | AA |  |  | BB |  |  |
|  | LPS 0 h | LPS 4 h | LPS 8 h | LPS 0 h | LPS 4 h | LPS 8 h |
| Zswim6 | 1.62 | 2.87 | 2.46 | 2.32 | 3.00 | 2.29 |
| Znrf3 | 0.45 | 0.61 | 0.43 | 0.37 | 0.88 | 0.46 |
| Zmym2 | 3.38 | 3.82 | 4.57 | 5.89 | 7.45 | 5.36 |
| Zfp704 | 0.33 | 0.08 | 0.24 | 0.46 | 0.29 | 0.11 |
| Zfp703 | 2.65 | 4.19 | 2.03 | 2.80 | 3.88 | 2.30 |
| Zfp644 | 8.53 | 10.32 | 11.28 | 11.93 | 14.12 | 11.51 |
| Zfp518b | 0.35 | 0.29 | 0.34 | 0.48 | 0.55 | 0.54 |
| Zfp518a | 1.61 | 2.19 | 3.82 | 2.78 | 5.37 | 4.56 |
| Zfp407 | 3.24 | 2.97 | 2.79 | 3.77 | 3.32 | 3.90 |
| Zfp236 | 3.67 | 6.45 | 5.60 | 7.57 | 11.77 | 8.17 |
| Ywhae | 143.80 | 411.46 | 370.14 | 261.56 | 397.40 | 439.21 |
| Wnk1 | 84.81 | 158.57 | 158.64 | 115.89 | 315.35 | 120.20 |
| Wee1 | 6.25 | 5.14 | 6.46 | 7.71 | 8.54 | 7.02 |
| Ust | 0.49 | 0.40 | 0.52 | 0.80 | 0.46 | 0.75 |
| Usp48 | 58.77 | 49.38 | 31.81 | 57.41 | 48.16 | 60.44 |
| Usp45 | 4.01 | 3.78 | 5.51 | 6.16 | 8.57 | 4.62 |
| Usp14 | 32.45 | 49.12 | 58.71 | 41.29 | 67.13 | 50.74 |
| Ttl | 1.90 | 0.95 | 1.78 | 1.29 | 1.59 | 2.27 |
| Ttc28 | 2.73 | 0.57 | 1.10 | 2.60 | 1.04 | 0.69 |
| Tshz3 | 0.70 | 0.34 | 0.42 | 0.63 | 0.40 | 0.85 |
| Trps1 | 0.49 | 0.56 | 0.63 | 0.82 | 0.94 | 0.64 |
| Trp53inp1 | 7.43 | 7.33 | 10.97 | 10.89 | 22.26 | 13.27 |
| Trip12 | 8.36 | 13.48 | 14.38 | 13.79 | 35.93 | 16.40 |
| Trim44 | 10.39 | 16.01 | 12.80 | 10.54 | 27.88 | 19.43 |
| Trim32 | 2.35 | 3.61 | 2.83 | 2.55 | 2.30 | 3.80 |
| Trim2 | 0.42 | 0.35 | 1.37 | 0.90 | 0.53 | 0.30 |
| Traf3 | 4.99 | 5.83 | 7.51 | 5.97 | 11.15 | 6.67 |
| Tm9sf3 | 12.03 | 15.20 | 16.83 | 13.31 | 22.60 | 18.21 |
| Tle4 | 3.25 | 3.24 | 3.10 | 4.40 | 6.09 | 2.87 |
| Thoc1 | 9.63 | 24.49 | 14.99 | 11.23 | 18.97 | 17.11 |
| Tfdp2 | 32.56 | 23.00 | 55.50 | 72.64 | 109.60 | 38.80 |
| Tcf7l2 | 2.72 | 1.11 | 2.34 | 2.84 | 4.46 | 0.98 |
| Tcf4 | 3.72 | 8.56 | 14.23 | 8.51 | 27.04 | 13.82 |
| Tada2b | 5.07 | 5.68 | 4.90 | 5.44 | 6.19 | 5.51 |
| Tab2 | 39.80 | 69.63 | 76.77 | 58.61 | 117.80 | 75.60 |
| Syvn1 | 30.03 | 18.50 | 10.79 | 21.19 | 18.70 | 16.55 |
| Sypl | 9.88 | 14.77 | 16.42 | 12.34 | 18.70 | 16.68 |
| Stx16 | 14.77 | 10.02 | 8.16 | 12.65 | 15.84 | 9.18 |
| Strn3 | 11.26 | 13.22 | 13.14 | 14.83 | 18.13 | 16.07 |
| Spty2d1 | 4.68 | 8.16 | 8.21 | 7.03 | 16.47 | 8.96 |
| Sp3 | 19.25 | 19.82 | 20.87 | 32.79 | 46.49 | 24.47 |
| Sp1 | 7.34 | 8.81 | 10.25 | 14.03 | 44.84 | 20.75 |
| Sos1 | 1.92 | 5.49 | 5.71 | 2.69 | 9.86 | 4.75 |
| Socs6 | 1.95 | 3.06 | 4.14 | 2.82 | 6.52 | 4.60 |
| Smug1 | 0.99 | 1.08 | 1.01 | 0.80 | 1.18 | 0.98 |
| Smarca4 | 45.45 | 33.22 | 36.94 | 43.82 | 38.34 | 35.23 |
| Smad5 | 2.32 | 3.05 | 6.88 | 4.76 | 8.21 | 6.43 |
| Sla | 29.18 | 44.02 | 62.40 | 43.04 | 82.29 | 57.95 |
| Ski | 6.74 | 3.96 | 4.74 | 6.77 | 3.88 | 5.43 |
| Sgk3 | 11.01 | 7.35 | 6.55 | 13.20 | 17.86 | 7.89 |
| Sfpi1 | 224.69 | 405.34 | 338.51 | 165.57 | 229.96 | 247.97 |
| Sema5a | 1.39 | 1.56 | 0.44 | 1.41 | 1.23 | 0.54 |
| Sdcbp | 74.46 | 101.81 | 111.94 | 80.49 | 176.74 | 110.51 |
| Sdcbp | 9.90 | 6.96 | 2.07 | 11.28 | 20.72 | 4.38 |
| Satb1 | 11.88 | 59.00 | 38.08 | 17.98 | 72.06 | 29.94 |
| S1pr1 | 34.66 | 43.45 | 37.49 | 45.15 | 30.54 | 41.44 |
| Rufy2 | 4.00 | 5.36 | 4.21 | 6.31 | 5.11 | 6.51 |
| Rsf1 | 11.31 | 15.67 | 11.62 | 14.35 | 15.66 | 12.38 |
| Rreb1 | 4.74 | 2.33 | 4.03 | 6.00 | 4.70 | 4.15 |
| Rps6ka5 | 1.34 | 0.73 | 1.67 | 2.40 | 1.33 | 1.51 |
| Rnf146 | 17.58 | 17.71 | 16.66 | 21.20 | 35.85 | 23.92 |
| Rnf123 | 265.61 | 97.41 | 72.10 | 211.01 | 152.31 | 100.64 |
| Rhoq | 4.73 | 6.27 | 8.13 | 6.40 | 9.65 | 7.97 |
| Rheb | 29.16 | 59.70 | 48.58 | 41.48 | 53.39 | 50.03 |
| Rgp1 | 8.74 | 4.08 | 4.51 | 8.44 | 10.93 | 5.51 |
| Reps2 | 0.32 | 0.26 | 0.17 | 0.58 | 0.56 | 0.18 |
| Rela | 33.47 | 53.99 | 31.33 | 23.51 | 34.20 | 27.67 |
| Rcn2 | 4.19 | 6.01 | 7.77 | 6.81 | 9.19 | 7.61 |
| Rab34 | 7.58 | 4.76 | 5.98 | 7.61 | 4.76 | 8.42 |
| Rab11fip2 | 1.07 | 1.52 | 2.27 | 1.74 | 1.71 | 1.96 |
| Qk | 10.99 | 16.58 | 18.35 | 19.49 | 38.86 | 19.90 |
| Ptprj | 7.76 | 9.78 | 9.79 | 11.91 | 18.27 | 10.46 |
| Ptpn2 | 31.97 | 155.46 | 118.06 | 33.55 | 87.46 | 110.33 |
| Pskh1 | 6.59 | 4.99 | 5.28 | 6.55 | 5.52 | 6.04 |
| Prpf39 | 7.96 | 13.90 | 12.25 | 11.56 | 21.25 | 13.68 |
| Ppil2 | 62.33 | 45.60 | 50.53 | 71.77 | 53.47 | 57.93 |
| Ppig | 17.20 | 51.66 | 74.31 | 39.20 | 93.93 | 66.10 |
| Plekhm1 | 11.86 | 10.23 | 9.88 | 10.16 | 7.70 | 10.05 |
| Plag1 | 0.09 | 0.09 | 0.33 | 0.35 | 0.39 | 0.34 |
| Pkn2 | 22.52 | 23.90 | 38.38 | 24.65 | 72.27 | 33.80 |
| Pkia | 1.69 | 0.82 | 0.83 | 1.60 | 0.67 | 0.94 |
| Phf21b | 1.94 | 0.19 | 0.30 | 3.00 | 2.09 | 1.48 |
| Phf17 | 3.73 | 3.48 | 4.63 | 4.89 | 4.92 | 4.71 |
| Pea15a | 31.38 | 17.11 | 13.63 | 27.69 | 17.65 | 17.75 |
| Pde7a | 23.94 | 20.53 | 16.88 | 32.50 | 34.37 | 20.10 |
| Pard3b | 0.49 | 0.61 | 1.42 | 0.98 | 0.89 | 0.64 |
| Pam | 9.58 | 23.16 | 22.91 | 13.95 | 20.61 | 16.10 |
| Nufip2 | 2.23 | 3.28 | 3.13 | 4.30 | 15.66 | 4.62 |
| Nova1 | 0.07 | 0.06 | 0.04 | 0.09 | 0.04 | 0.06 |
| Nfia | 0.61 | 0.16 | 0.29 | 0.66 | 0.88 | 0.56 |
| Nfe2l2 | 69.79 | 131.21 | 99.42 | 86.55 | 94.17 | 107.03 |
| Nfat5 | 3.13 | 4.56 | 3.35 | 8.43 | 28.27 | 4.98 |
| N4bp1 | 6.62 | 22.49 | 21.01 | 7.07 | 29.01 | 20.44 |
| Myo1d | 7.12 | 6.61 | 5.79 | 7.35 | 10.32 | 4.50 |
| Myo10 | 2.21 | 5.74 | 7.53 | 4.46 | 7.45 | 4.95 |
| Myb | 7.94 | 10.55 | 11.33 | 14.94 | 17.51 | 11.40 |
| Mxi1 | 5.75 | 2.49 | 4.16 | 6.52 | 6.85 | 3.50 |
| Ms4a1 | 104.65 | 103.47 | 44.85 | 122.00 | 77.06 | 73.45 |
| Mon1a | 9.70 | 8.48 | 4.94 | 8.41 | 5.41 | 5.35 |
| Mlycd | 6.14 | 2.84 | 4.41 | 5.24 | 2.06 | 2.94 |
| Midn | 11.78 | 6.03 | 4.19 | 12.52 | 13.65 | 6.90 |
| Mid2 | 0.77 | 0.54 | 0.75 | 1.03 | 0.97 | 0.56 |
| Mex3b | 0.94 | 0.33 | 0.38 | 1.29 | 0.84 | 0.56 |
| Meis1 | 2.85 | 2.61 | 0.90 | 4.05 | 1.83 | 1.82 |
| Mef2a | 7.01 | 14.27 | 10.78 | 10.42 | 13.37 | 11.88 |
| Mbtd1 | 3.02 | 6.41 | 6.94 | 3.83 | 11.84 | 7.71 |
| Mark2 | 66.96 | 51.49 | 45.22 | 72.72 | 57.52 | 35.70 |
| Map3k10 | 3.00 | 2.16 | 0.93 | 3.03 | 1.39 | 1.88 |
| Mafb | 5.17 | 2.52 | 4.23 | 5.38 | 3.48 | 3.29 |
| Lrrc59 | 53.77 | 105.65 | 93.22 | 50.83 | 71.41 | 87.10 |
| Lekr1 | 0.15 | 1.13 | 0.15 | 0.36 | 0.13 | 0.24 |
| Kras | 19.73 | 30.96 | 38.72 | 27.95 | 43.86 | 27.74 |
| Kpna4 | 5.09 | 9.88 | 8.68 | 7.87 | 15.35 | 10.67 |
| Kpna1 | 7.11 | 11.97 | 13.49 | 18.06 | 34.68 | 10.19 |
| Kdm2a | 20.15 | 13.52 | 19.32 | 29.89 | 38.96 | 28.88 |
| Jhdm1d | 5.70 | 3.86 | 7.59 | 9.13 | 16.44 | 7.98 |
| Jarid2 | 4.22 | 5.69 | 7.33 | 6.82 | 11.51 | 5.89 |
| Itk | 6.07 | 13.68 | 8.42 | 6.73 | 15.42 | 10.83 |
| Itgb1bp1 | 33.66 | 40.38 | 41.89 | 29.60 | 28.12 | 43.77 |
| Irf2bp2 | 2.17 | 3.10 | 3.25 | 2.27 | 4.77 | 2.19 |
| Inpp5d | 36.07 | 14.49 | 24.39 | 34.44 | 18.94 | 20.64 |
| Inadl | 2.26 | 2.70 | 3.30 | 3.06 | 1.91 | 3.08 |
| Il21 | 0.19 | 0.54 | 0.07 | 0.28 | 0.56 | 0.23 |
| Ikbke | 15.80 | 61.71 | 59.94 | 15.45 | 53.22 | 58.91 |
| Hivep2 | 2.85 | 3.54 | 3.51 | 3.50 | 7.01 | 4.96 |
| Hbp1 | 4.84 | 3.40 | 7.32 | 8.73 | 4.55 | 38.30 |
| Hbp1 | 34.70 | 43.66 | 44.53 | 9.32 | 4.41 | 8.52 |
| Hbp1 | 11.25 | 30.16 | 12.50 | 11.97 | 7.96 | 10.22 |
| Hbp1 | 14.80 | 14.48 | 15.66 | 17.96 | 17.24 | 19.68 |
| Hbp1 | 5.80 | 10.28 | 4.92 | 16.52 | 15.09 | 16.42 |
| Hbp1 | 8.05 | 7.85 | 5.16 | 43.96 | 51.69 | 45.98 |
| H3f3a | 295.89 | 127.27 | 142.88 | 282.36 | 306.19 | 140.47 |
| Gsk3b | 3.04 | 6.25 | 6.86 | 6.74 | 10.01 | 8.75 |
| Gpm6b | 4.53 | 4.17 | 5.02 | 4.91 | 6.73 | 6.88 |
| Gpd1l | 9.80 | 4.51 | 4.94 | 10.10 | 5.51 | 7.05 |
| Gm12657 | 2.29 | 2.74 | 2.71 | 1.44 | 1.77 | 2.16 |
| Gdf6 | 2.16 | 0.10 | 0.14 | 2.61 | 0.34 | 0.27 |
| Fzd5 | 0.55 | 0.85 | 0.87 | 0.73 | 1.90 | 0.76 |
| Foxk1 | 3.24 | 3.10 | 2.86 | 3.55 | 4.94 | 3.48 |
| Fosl2 | 1.23 | 6.15 | 4.09 | 1.29 | 8.61 | 5.26 |
| Fgf7 | 0.29 | 1.31 | 1.21 | 1.40 | 1.38 | 0.47 |
| Fbxo33 | 6.17 | 6.48 | 5.99 | 12.95 | 18.70 | 8.09 |
| Fbxo11 | 12.51 | 27.28 | 21.60 | 17.77 | 33.16 | 22.09 |
| Fbxl2 | 1.42 | 2.27 | 2.17 | 6.50 | 2.48 | 2.66 |
| Fbxl2 | 4.78 | 1.94 | 2.12 | 4.62 | 8.34 | 3.61 |
| Far1 | 12.62 | 18.30 | 19.79 | 24.83 | 64.29 | 24.64 |
| Fam84a | 0.47 | 0.31 | 0.25 | 0.57 | 0.34 | 0.20 |
| Fam168a | 5.45 | 2.65 | 4.64 | 7.83 | 4.08 | 6.15 |
| Fam135a | 0.54 | 0.51 | 0.76 | 0.56 | 0.63 | 0.70 |
| Fam105a | 29.44 | 6.90 | 3.60 | 37.55 | 10.67 | 11.26 |
| Etv3 | 2.91 | 8.71 | 7.12 | 4.01 | 18.11 | 6.86 |
| Ets1 | 23.83 | 19.28 | 19.52 | 32.11 | 34.08 | 26.90 |
| Entpd7 | 0.49 | 0.35 | 0.46 | 0.54 | 0.52 | 0.53 |
| Emp2 | 3.45 | 1.24 | 1.80 | 3.11 | 1.47 | 2.11 |
| Ell2 | 15.10 | 17.79 | 13.97 | 18.72 | 26.79 | 19.76 |
| Ehf | 0.68 | 2.77 | 2.41 | 1.51 | 6.87 | 0.79 |
| Eefsec | 10.78 | 13.36 | 10.37 | 8.84 | 7.72 | 8.04 |
| E2f2 | 37.53 | 12.59 | 39.03 | 31.43 | 53.92 | 30.01 |
| Dync1i1 | 0.40 | 0.11 | 0.13 | 0.20 | 0.21 | 0.27 |
| Dusp7 | 9.82 | 8.07 | 6.65 | 10.32 | 11.45 | 6.46 |
| Dnajb1 | 29.14 | 13.04 | 13.82 | 23.28 | 21.23 | 12.44 |
| Dmtf1 | 14.16 | 24.38 | 23.24 | 15.89 | 19.74 | 22.92 |
| Dirc2 | 5.81 | 4.49 | 4.18 | 4.41 | 4.45 | 4.06 |
| Dhx40 | 6.68 | 6.17 | 10.71 | 8.35 | 10.10 | 8.27 |
| Det1 | 6.31 | 7.40 | 4.39 | 7.30 | 6.26 | 5.49 |
| Dcun1d3 | 1.65 | 1.60 | 1.41 | 2.45 | 3.05 | 1.70 |
| D15Ertd621e | 4.89 | 6.83 | 7.92 | 6.53 | 12.17 | 10.71 |
| Cyp7b1 | 0.53 | 3.71 | 2.29 | 0.70 | 4.35 | 1.79 |
| Cyb561d1 | 2.27 | 1.72 | 2.30 | 2.67 | 2.21 | 2.27 |
| Cux1 | 35.79 | 37.15 | 34.58 | 43.16 | 31.90 | 30.94 |
| Csnk1g2 | 58.86 | 33.59 | 31.55 | 50.34 | 22.77 | 35.38 |
| Csf1r | 121.83 | 41.29 | 29.15 | 96.53 | 28.63 | 22.30 |
| Creb1 | 4.38 | 6.33 | 5.54 | 6.97 | 12.03 | 7.64 |
| Cntln | 3.94 | 2.77 | 3.07 | 3.76 | 3.27 | 4.00 |
| Cds1 | 1.08 | 1.19 | 0.84 | 1.11 | 2.03 | 1.78 |
| Cd99l2 | 4.24 | 0.93 | 1.94 | 4.83 | 2.69 | 2.13 |
| Ccbe1 | 0.49 | 0.38 | 0.99 | 0.55 | 1.01 | 1.18 |
| Card11 | 46.47 | 12.06 | 10.17 | 37.52 | 8.10 | 13.99 |
| Camta1 | 9.91 | 17.01 | 13.55 | 15.70 | 17.47 | 13.75 |
| Cacna2d1 | 0.22 | 0.63 | 0.96 | 0.45 | 0.82 | 0.52 |
| Cacna1c | 0.17 | 0.14 | 0.10 | 0.19 | 0.27 | 0.15 |
| Cab39 | 16.11 | 21.38 | 25.21 | 20.04 | 52.18 | 27.45 |
| Brd1 | 9.15 | 10.41 | 11.77 | 12.44 | 21.37 | 11.16 |
| Boc | 0.58 | 0.38 | 0.70 | 0.65 | 0.31 | 0.66 |
| Baiap2l1 | 0.31 | 0.28 | 1.15 | 0.15 | 0.64 | 0.57 |
| Bach1 | 5.02 | 10.85 | 7.06 | 6.02 | 14.48 | 7.61 |
| Atxn1l | 1.63 | 1.56 | 1.80 | 1.66 | 3.95 | 2.25 |
| Atp6v1g1 | 31.07 | 33.51 | 42.15 | 33.24 | 41.98 | 35.24 |
| Atg3 | 28.99 | 39.50 | 37.85 | 29.22 | 40.84 | 36.17 |
| Astn2 | 2.70 | 1.95 | 1.16 | 1.83 | 0.95 | 1.24 |
| Arrb2 | 138.52 | 81.00 | 133.67 | 102.72 | 50.31 | 94.49 |
| Arl5b | 0.70 | 1.68 | 1.38 | 1.17 | 4.81 | 1.38 |
| Arid4a | 9.92 | 7.92 | 10.04 | 16.14 | 20.57 | 11.32 |
| Apc | 2.71 | 3.08 | 3.00 | 4.46 | 10.39 | 3.50 |
| Aicda | 1.79 | 1.69 | 0.55 | 1.33 | 0.88 | 0.75 |
| Agap1 | 1.87 | 0.52 | 0.82 | 1.88 | 0.85 | 0.94 |
| Adam10 | 28.20 | 20.01 | 28.59 | 41.39 | 51.99 | 27.80 |
| Acvr2a | 0.65 | 1.05 | 2.59 | 0.79 | 3.49 | 1.54 |
| Actr10 | 4.70 | 5.38 | 5.73 | 4.52 | 7.21 | 5.71 |
| Aak1 | 3.30 | 0.39 | 1.16 | 4.20 | 9.77 | 2.28 |
| 2610002M06Rik | 3.07 | 4.27 | 4.70 | 3.77 | 7.94 | 5.29 |
| 2510009E07Rik | 1.38 | 1.00 | 1.46 | 1.91 | 1.64 | 1.41 |
| 1810011O10Rik | 2.44 | 0.24 | 0.56 | 2.64 | 0.18 | 0.62 |
