# Supplementary material for: Natural Functional SNPs in miR-155 Alter Its Expression Level, Blood Cell Counts, and Immune Responses
Source: Front Immunol. 2016 Aug 2;7:295. doi: 10.3389/fimmu.2016.00295 (PMC4970381; doi:10.3389/fimmu.2016.00295)
Supplement: Supplementary file 6 [file image_1.pdf]

A GCAGGTGCTGCAAACCAGGAAGGGGAAGTGTGTGGTTTAAGTTGCATATCCCTT
B GCAGGTGCTGCAAACCAGGAAGGGGAAGTGTGTGGTTTAAGTTGCATATCCCTT

A ATCCTCTGCCTGCTGGAGGCTTGCTGAAGGCTGTATGCTGTTAATGCTAATTGTG
B ATCCTCTGGCTGCTGGAGGCTTGCTGAAGGCTGTATGCTGTTAATGCTAATTGTG

mmu-miR155

A ATAGGGGTTTTGGCGTCTGACTGACTCCTACCTGTTAGCATTAACAGGACACAAC
B ATAGGGGTTTTGGCCTCTGACTGACTCCTACCTGTTAGCATTAACAGGACACAAG

Stem-loop mmu-miR155*

A GCCTGTTACTAGCACTCACATGGAACAAATGGCCACCGTGGGAGGATGACAAGT
B GCCTGTTACTAGCACTCACATGGAACAAATGGCCACCGTGGGAGGATGACAAGT

A CCAAGAGTCACTCTGCTGGATGAACGTAGATGTCAGAC
B CCAAGAGTCACCCTGCTGGATGAACGTAGATGTCAGAC

Supplementary figure 1. The sequence of the A and B haplotype of mouse miR-155.
