# Supplementary material for: Natural Functional SNPs in miR-155 Alter Its Expression Level, Blood Cell Counts, and Immune Responses
Source: Front Immunol. 2016 Aug 2;7:295. doi: 10.3389/fimmu.2016.00295 (PMC4970381; doi:10.3389/fimmu.2016.00295)
Supplement: Supplementary file 7 [file image_2.pdf]

h CAGGTGGCACAAACCAGGAAGGGGAAATCTGTGGTTTAAATTCTTTATGCCTCA
h1 CAGGTGGCACAAACCAGGAAGGGGAAATCTGTGGTTTAAATTCTTTATGCCTCA
h2 CAGGTGGCACAAACCAGGAAGGGGAAATCTGTGGTTTAAATTCTTTATGCCTCA
h3 CAGGTGGCACAAACCAGGAAGGGGAAATCTGTGGTTTAAATTCTTTATGCCTCA
h4 CAGGTGGCACAAACCAGGAAGGGGAAATCTGTGGTTTAAATTCTTTATGCCTCA

h TCCTCTGAGTGCTGAAGGCTTGCTGTAGGCTGTATGCTGTTAATGCTAATCGTGA
h1 TCCTCTGAGTGCTGAAGGCTTGCTGTAGGCTGTATGCTGTTAATGCTAATCATGA
h2 TCCTCTGAGTGCTGAAGGCTTGCTGTAGGCTGTATGCTGTTAATGCTAATCGTGA
h3 TCCTCTGAGTGCTGAAGGCTTGCTGTAGGCTGTATGCTGTTAATGCTAATCGTGA
h4 TCCTCTGAGTGCTGAAGGCTTGCTGTAGGCTGTATGCTGTTAATGCTAATCGTGA

hsa-miR155

h TAGGGGTTTTTGCCTCCAACTGACTCCTACATATTAGCATTAACAGTGTATGATGC
h1 TAGGGGTTTTTGCCTCCAACTGACTCCTACATATTAGCATTAACAGTGTATGATGC
h2 TAGGGGTTTTTGCCCCCAACTGACTCCTACATATTAGCATTAACAGTGTATGATGC
h3 TAGGGGTTTTTGCCTCCAACTGACTCCTACGTATTAGCATTAACAGTGTATGATGC
h4 TAGGGGTTTTTGCCTCCAACTGACTCCTACATGTTAGCATTAACAGTGTATGATGC

stem loop hsa-miR155*

h CTGTTACTAGCATTCACATGGAACAAATTGCTGCCGTGGGAGGATGACAAAGAAG
h1 CTGTTACTAGCATTCACATGGAACAAATTGCTGCCGTGGGAGGATGACAAAGAAG
h2 CTGTTACTAGCATTCACATGGAACAAATTGCTGCCGTGGGAGGATGACAAAGAAG
h3 CTGTTACTAGCATTCACATGGAACAAATTGCTGCCGTGGGAGGATGACAAAGAAG
h4 CTGTTACTAGCATTCACATGGAACAAATTGCTGCCGTGGGAGGATGACAAAGAAG

h CATGAGTCACCCTGCTGGATAAACTTAGACTTCAG
h1 CATGAGTCACCCTGCTGGATAAACTTAGACTTCAG
h2 CATGAGTCACCCTGCTGGATAAACTTAGACTTCAG
h3 CATGAGTCACCCTGCTGGATAAACTTAGACTTCAG
h4 CATGAGTCACCCTGCTGGATAAACTTAGACTTCAG

Supplementary figure 2. The sequence of the mutants of human miR-155.
