# Supplementary material for: Natural Functional SNPs in miR-155 Alter Its Expression Level, Blood Cell Counts, and Immune Responses
Source: Front Immunol. 2016 Aug 2;7:295. doi: 10.3389/fimmu.2016.00295 (PMC4970381; doi:10.3389/fimmu.2016.00295)
Supplement: Supplementary file 8 [file image_3.pdf]

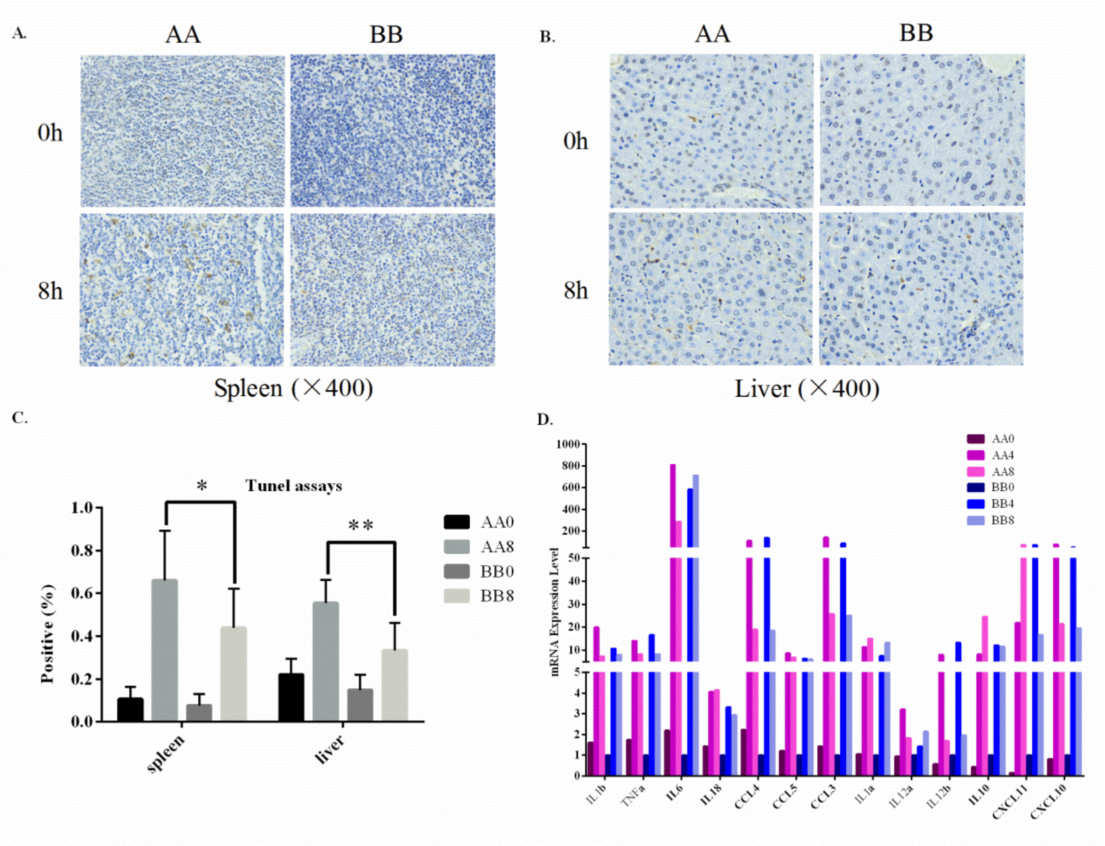

Supplementary figure 3. The inflammatory responses in the AA genotype Kunming mice were stronger than that in BB genotype Kunming mice under LPS treatment.

(A-B) Apoptosis in the spleen and liver tissues at 0 and 8 hours of LPS exposure was detected by using TUNEL assay. scale bar=50 μm. (C) The proportion of apoptotic cells in the spleen and liver tissues of AA genotype mice was significantly higher than that of BB genotype mice at 8 hours of LPS exposure. At least 8 visual fields of each tissue were chosen randomly for statistical analysis. The results are presented as the means ± S.E.M. ($n \geq 8$). (D) The mRNA levels of the cytokines and chemokines in the spleen tissues of AA and BB genotype of Kunming mice based on the RNA-seq data. The transcriptional levels of all the genes at 4 or 8 hours of LPS exposure were up-regulated compared to that at 0 hours. The transcriptional levels of IL1β, TNFα, IL6, IL1α, IL18, CCL4, CCL5 and CCL3 genes were relatively higher in the AA genotype mice than in the BB genotype mice at 0 hours. Whereas, the transcriptional levels of CXCL11, IL10 and IL12b genes were relatively higher in the BB genotype mice compared to that in the AA genotype mice at 0 hours. The mRNA level of each gene at 0 hours in BB genotype mice was normalized to 1. *$P<0.05$; ** $P<0.01$.
